# Computational multiscale analysis of growth and remodeling in the ascending aorta of the Marfan mouse

**DOI:** 10.1007/s10237-026-02134-x

**Published:** 2026-10-07

**Authors:** Ruturaj M. Badal, Intekhab Alam, Lily R. Watkins, Krashn K. Dwivedi, Jessica E. Wagenseil, Victor H. Barocas

**Affiliations:** 1 Department of Mechanical Engineering, University of Minnesota – Twin Cities, Minneapolis, MN USA; 2 Department of Mechanical Engineering and Materials Science, Washington University in St. Louis, St. Louis, MO USA; 3 Department of Aerospace Engineering and Mechanics, University of Minnesota – Twin Cities, Minneapolis, MN USA; 4 Department of Biomedical Engineering, University of Minnesota – Twin Cities, Minneapolis, MN USA

**Keywords:** Biomechanics, Aorta, Aneurysm

## Abstract

Marfan syndrome, caused primarily by mutations in the fibrillin-1 (*FBN1*) gene, is associated with progressive dilation of the proximal aorta, with dissection and rupture representing the leading causes of early mortality. A characteristic feature of Marfan-associated aneurysm is fragmentation of elastic fibers; however, the extent to which elastin loss alone can account for the observed geometric and mechanical changes remains unclear. In this study, we use a multiscale computational growth and remodeling framework to determine which geometric and mechanical features of the Marfan aorta can be reproduced by prescribed elastin loss acting through mechanically regulated fiber remodeling and tissue growth. The model couples a discrete fiber network representation of collagen, elastin, and actomyosin with a continuum description of the tissue. It is first calibrated to reproduce experimental measurements in the healthy mouse aorta, and Marfan conditions are then introduced by prescribing graded reductions in elastin content (10–80%). The model reproduces key features of the Marfan aorta, including increased wall thickness and radius, reduced axial stress and stretch, and shifts in pressure-dependent stiffness. Quantitative comparison across multiple metrics shows strong agreement between simulations and experiments, with the lowest error occurring at 29% elastin loss. Within the scope of the model, prescribed elastin loss led to many key structural and mechanical features observed in Marfan aortas, demonstrating the predictive potential of the framework for exploring the mechanical consequences of elastin fragmentation.

**Supplementary Information:**

The online version contains supplementary material available at https://doi.org/10.1007/s10237-026-02134-x.

## Introduction

Marfan syndrome, an inherited connective tissue disorder caused primarily by mutations in the fibrillin-1 (*FBN1*) gene, affects roughly one in 5000 individuals (Dietz et al. 1991; Judge and Dietz 2005). Although other tissues are also affected, progressive dilation of the proximal aorta and the high incidence of aortic dissection or rupture remain the major causes of early mortality in untreated patients with Marfan syndrome (Pyeritz 2000; Milewicz et al. 2021). As a result, understanding how Marfan syndrome perturbs aortic structure and mechanics is essential for forecasting disease progression and for developing more targeted treatment strategies (Isselbacher et al. 2022).

Elastin normally exhibits extraordinary longevity, with minimal turnover during adulthood (Shapiro et al. 1991). Yet, fragmentation of elastic fibers is a characteristic lesion in Marfan-syndrome-associated aneurysms (Wanga et al. 2017). Given that *FBN1* supports the assembly and durability of the elastin–microfibril network (Davis 1993), pathogenic *FBN1* variants presumably undermine this scaffold and drive a progressive loss of elastic fiber integrity. Aberrant vascular smooth muscle cell phenotypes, dysregulated transforming growth factor-β (TGF-β) signaling, and elevated matrix metalloproteinase activity have been identified as key contributors to this process (Seeburun et al. 2023). Much of what we know about the downstream consequences of this degradation comes from mouse models of Marfan syndrome, where changes in thoracic aortic histology and biomechanics have been quantified extensively (e.g., Marque et al. 2001; Bellini et al. 2016). Recent investigations (Cavinato et al. 2021; Tarraf et al. 2024; Dwivedi et al. 2024, 2025) have provided a comprehensive picture of how the defects in Marfan-affected mice evolve.

Computer modeling, which has advanced considerably in recent years, offers a potential tool to study Marfan syndrome. Both discrete and continuum frameworks have been developed, each capturing complementary aspects of multiscale arterial adaptation (Rodriguez et al. 1994; Taber 1998; Humphrey and Rajagopal 2002). Hybrid methods that couple discrete microscale behavior with continuum tissue-level mechanics have further expanded our ability to interrogate complex vascular phenomena (Braeu et al. 2017; Flanary et al. 2023). In a recent work, Li et al. (2023) leveraged the constrained mixture model to study aneurysm progression in Marfan syndrome and concluded that elastin loss alone was not sufficient to explain the observed vessel geometry and mechanical changes. In previous work, we developed a multiscale remodeling model capable of reproducing ex vivo arterial responses under controlled loading (Badal et al. 2025). Because Marfan syndrome alters both structure and function, a unified framework incorporating both growth and remodeling can complement experimental studies in testing the hypothesis about disease progression.

We therefore asked whether prescribed elastin loss, acting through fiber-scale mechanically regulated remodeling, could reproduce key geometric and mechanical features of the Marfan ascending aorta. In this study, to test that hypothesis, we first established the multiscale computational growth and remodeling framework to analyze the ascending aorta in Marfan syndrome, and we calibrated the model against experimental geometric and mechanical measurements reported by Dwivedi et al. (2025) for a severe Marfan mouse model. Using this calibrated baseline configuration to represent the wild-type state, elastin loss was imposed as a prescribed perturbation within the fiber-scale representation of the aortic wall. By systematically varying the magnitude of elastin loss, we evaluated how reductions in elastin content influence geometric, mechanical, and microstructural outcomes predicted by the model.

Although Marfan syndrome induces abnormalities throughout the arterial tree, the ascending thoracic aorta, which has the highest elastin content in the aorta (Concannon et al. 2020; Liyanage et al. 2022) exhibits the most pronounced disruption of matrix organization and passive mechanics and is the segment most prone to aneurysmal degeneration. For these reasons, our analysis focused exclusively on the ascending thoracic aorta. Although biochemical signaling undoubtedly contributes to disease progression (Irons and Humphrey 2020), the current study isolates the mechanical consequences of elastin loss, aiming to provide a bridge between microstructural degradation and macroscopic aortic pathology.

## Methods

### Formulation

We use a discrete fiber network model, as we have done previously (Gacek et al. 2022; Mahutga et al. 2025; Badal et al. 2025). Because the model has been described in considerable detail elsewhere, we provide here a basic description focusing on the key features and changes from previous versions, and we provide a highly detailed description of the methods in the Supplementary Material. The model can be viewed as a constrained mixture of a discrete fiber network (DFN) and a neo-Hookean (NH) solid matrix. The detailed structure of the model allows it to account for non-affine interaction of tissue components, as well as the complex microarchitecture of the aortic media, in multiple ways. First, it captures the tissue’s compositional heterogeneity at the small scale, with a distinct collagen–elastin lamella separating regions of smooth muscle cells (represented as actomyosin fibers in the model). Second, the model can account for differential remodeling of different fibers under fiber-specific loads; that is, an individual heavily-loaded fiber can behave differently from a lightly-loaded fiber even if they have the same orientation.

Although the problem is geometrically simple enough that a single DFN could be used, it is computationally convenient to treat the problem as a multiscale model with one finite element in the macrodomain. The present approach follows the multiscale network plugin formulation of Mahutga et al. (2023), which enables direct incorporation of DFN into a continuum mechanics code. The methodology for constructing these DFNs followed our previously established approach (Gacek et al. 2022; Mahutga et al. 2023, 2025). Supplementary section S2 describes in detail how these networks are created. At the tissue scale, the material can be imagined as a constrained mixture of the DFN and an isotropic bulk matrix modeled as a compressible neo-Hookean solid,

1 $$\sigma ={\sigma }^{\mathrm{DFN}}+{\sigma }^{\mathrm{NH}}$$

where σ is the net Cauchy stress, ${\sigma }^{\mathrm{DFN}}$ is the Cauchy stress due to the DFN and ${\sigma }^{\mathrm{NH}}$ is the neo-Hookean contribution to the total Cauchy stress tensor.

### Mechanics

#### Discrete fiber networks

**Fig. 1:**
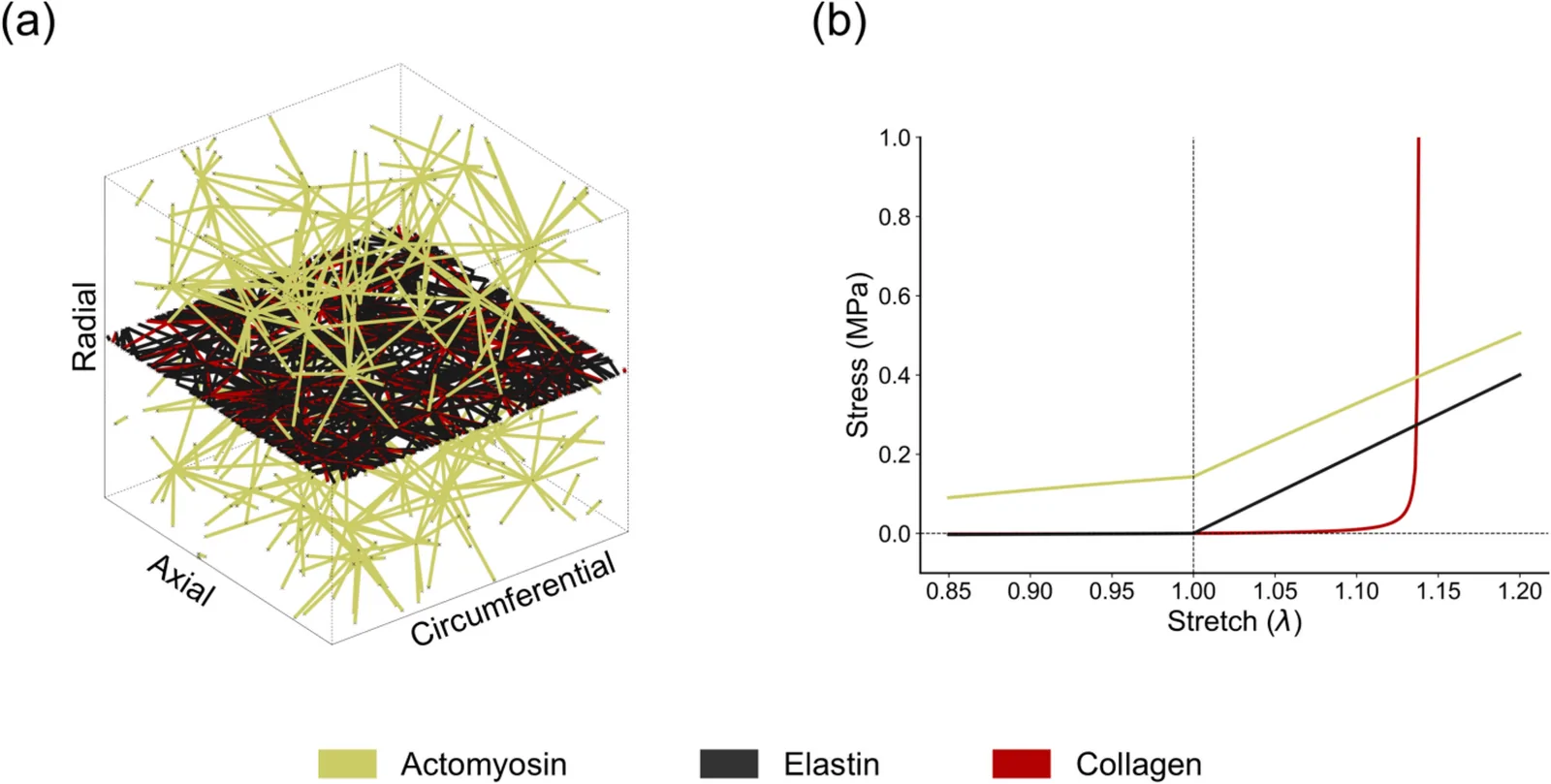
**a** Representative DFN of a medial lamellar unit showing elastin, collagen, and actomyosin fiber organization. **b** Constitutive stress–stretch behavior of individual fiber types used in the model

The three fiber systems in the medial lamellar unit—collagen, elastin, and actomyosin—were each assigned distinct constitutive models to reflect their biological and mechanical roles (Fig. 1). Collagen fibers were modeled using a nonlinear helical spring formulation (Freed and Doehring 2005), parameterized to align with experimental measurements of single fibrils (Flynn et al. 2010). The helical model accounts for the geometric parameters of fibril diameter, helix pitch, and coil radius. The resulting characteristic force–stretch curve is initially compliant but rapidly stiffens once the helix uncoils. Elastin fibers were represented as tension–compression asymmetric linear elastic elements, consistent with their propensity to buckle under compression due to high aspect ratio (Aaron and Gosline 1981). Although the molecular composition of elastic fibers includes elastin and > 30 associated proteins (Kielty 2006), we use the term “elastin fibers” here to indicate fibers mostly composed of elastin rather than the term “elastic fibers” which may be confused with fibers that behave elastically.

Actomyosin fibers were modeled as contractile structures with both active and passive components (Rachev and Hayashi 1999). Actomyosin fibers should be interpreted as a coarse-grained representation of the cellular/contractile smooth muscle component of the medial lamellar unit, rather than as a literal independent extracellular fiber network. This modeling choice allows smooth muscle contractility, remodeling, and growth to be incorporated within the DFN framework, but does not explicitly resolve individual cells, cell membranes, focal adhesions, or transmembrane protein-mediated coupling between smooth muscle cells and the surrounding extracellular matrix. For actomyosin, the total fiber force was defined as the sum of active and passive components, where the passive component follows the same bilinear relation as elastin, and the active component is stretch-dependent. The supplementary methods section explains in detail the mechanical response of each individual component with equations. Material parameters for all three fiber types were drawn from experimental literature and are summarized in Table 1. In this approach, the stress contribution of the fiber network is obtained through volume averaging, following Chandran and Barocas (2007) and Stylianopoulos and Barocas (2007). The average DFN Cauchy stress, ${\sigma }^{\mathrm{DFN}}$, within a representative volume V, is expressed as

2 $${\sigma }^{\mathrm{DFN}}=\frac{1}{V}{\sum_{e}}{f}^{e}{l}^{e}{n}^{e}\otimes {n}^{e}$$

where ${f}^{e}$ is the scalar force on fiber e, ${l}^{e}$ is the current fiber length, ${n}^{e}$ is the unit vector along the fiber direction in the deformed configuration, and V is the volume in the deformed configuration.

**Table 1:** Principal model parameters, sources, and experimental or modeling basis

Component or input	Parameter value	Source type	Source and original condition
Collagen fiber mechanics	${H}_{0} = 67.4\, \mathrm{n}\mathrm{m}$; ${R}_{0} = 5.8\, \mathrm{n}\mathrm{m}$; ${r}_{0} = 1.6\, \mathrm{n}\mathrm{m}$;$E = 700\, \mathrm{M}\mathrm{P}\mathrm{a}$	Experimental literature and constitutive model	Helical fibril formulation from Freed and Doehring (2005), with parameters adapted from type-I collagen fibril studies, including Flynn et al. (2010). These values were not measured directly in mouse aorta
Elastin fiber mechanics	$E = 2 \,\mathrm{M}\mathrm{P}\mathrm{a}$	Experimental literature	Based on mechanical measurements of isolated elastin fibers and elastic proteins (Aaron and Gosline 1981; Gosline et al. 2002). This value was not measured directly in mouse aorta
Actomyosin mechanics	${\lambda}_{\mathrm{m}\mathrm{a}\mathrm{x}} = 1.4$; ${\lambda}_{\mathrm{m}\mathrm{i}\mathrm{n}} = 0.65$; ${S}_{0} = 0.2\,\mathrm{M}\mathrm{P}\mathrm{a}$;$E = 1.6\, \mathrm{M}\mathrm{P}\mathrm{a}$	Literature-based phenomenological model	Adapted from the arterial smooth muscle contraction formulation of Rachev and Hayashi (1999). These are effective actomyosin parameters rather than direct measurements of intracellular fibers
Neo-Hookean bulk matrix	$\mu = 172.4 \,\mathrm{k}\mathrm{P}\mathrm{a}$; $\lambda = 1.55\,\mathrm{M}\mathrm{P}\mathrm{a}$	Previous modeling work	Adopted from the DFN-continuum formulation used in previous work from our group. These effective matrix parameters were not independently measured in the present mouse aortas
Actomyosin remodeling	$\tau = 4\, \mathrm{d}\mathrm{a}\mathrm{y}\mathrm{s}$; ${P}_{\infty } = 0.75\, \mathrm{M}\mathrm{P}\mathrm{a}$	Literature-informed previous modeling	Adopted from our previous remodeling model. The remodeling timescale was informed by experimental studies of vascular smooth muscle mechano-adaptation. The target stress is a phenomenological remodeling set point rather than a directly measured in vivo quantity
Collagen remodeling	$\tau = 90\, \mathrm{d}\mathrm{a}\mathrm{y}\mathrm{s}$; ${P}_{\infty } = 2\, \mathrm{M}\mathrm{P}\mathrm{a}$	Literature-informed previous modeling	Adopted from previous DFN remodeling studies. The remodeling timescale was informed by arterial collagen-turnover measurements. The target stress is a phenomenological remodeling set point rather than a direct measurement in Marfan mouse aorta
Elastin remodeling and prescribed loss	No normal turnover; radius reduction over $t = 90\, \mathrm{d}\mathrm{a}\mathrm{y}\mathrm{s}$; total elastin loss varied from $10-80{\%}$	Literature-informed assumption and study-specific prescription	The absence of normal elastin turnover was based on the long biological half-life of elastin. The timing of imposed loss was guided by the experimentally observed reduction and subsequent stabilization of elastin content in severe Marfan mice. The 10–80% loss range was varied parametrically
Wild-type calibration targets	$P \approx 105\, \mathrm{m}\mathrm{m}\mathrm{H}\mathrm{g}$; $h = 134 \pm 9.5\, \upmu \mathrm{m}$; $R = 447 \pm 29 \,\upmu \mathrm{m}$; ${\lambda}_{\mathrm{z}} = 1.79 \pm 0.26$;${\lambda}_{\uptheta } = 1.79 \pm 0.21$	Experimental data from the present study	Measurements from four-month-old male wild-type littermates in the severe *Fbn1mgR/mgR* mouse study. Additional stress and modulus measurements are reported in the experimental results table

#### Neo-Hookean

The compressible neo-Hookean material model as implemented by FEBio (Maas et al. 2012) is used for the bulk material. The strain energy density of the neo-Hookean matrix is given by

3 $${W}^{\mathrm{NH}}=\frac{\mu }{2}({I}_{1}-3)-\mu \hbox{ln}(J)+\frac{\lambda }{2}(\hbox{ln}J)^{2}$$

where μ and λ are the Lame-parameters, ${I}_{1}$ is the first invariant of the right Cauchy–Green tensor, and J is the determinant of the deformation gradient. In all models, we set $\mu =172.4\,\hbox{kPa}$ and $\lambda =1.55\,\hbox{MPa}$ (Mahutga et al. 2025), making the tissue nearly incompressible. The homogenized tissue stress is then obtained as the superposition of the matrix contribution and the volume-averaged stress of the DFN. In practice, this formulation permits direct coupling between fiber-level mechanics and tissue-level finite element solutions.

### Growth and remodeling framework

Experimental evidence describing how new mass is incorporated into, or removed from, the arterial wall remains limited (Grytsan et al. 2017). Mixture theory provides several plausible descriptions for deposition and removal (Eriksson et al. 2014). Under the constant constituent density assumption, a constituent’s mass can increase or decrease without altering its density; therefore, changes in constituent mass directly reflect changes in constituent volume fraction and/or the total material volume (Schmid et al. 2012). In contrast, the variable constituent density assumption allows mass to be added or removed while holding the constituent’s volume fixed, effectively changing its density rather than its geometric presence in the mixture. Here, we adopted a hybrid approach that applies constant constituent density to some fiber families and variable constituent density to others, following prior constrained mixture formulations.

#### Tissue volume

Because different structural components of the arterial wall remodel through distinct biological pathways, we used different assumptions to connect constituent-level remodeling to tissue-level volume change. Actomyosin, representing the cellular/smooth muscle component, was modeled using a constant density formulation. This choice was motivated by the role of smooth muscle as a major volume contributing component of the ascending aortic wall (Harkness et al. 1957; Apter et al. 1966; Badal and Barocas 2025). In this formulation, changes in actomyosin content directly contribute to the growth tensor and therefore to tissue-level volumetric growth.

Collagen was modeled using a variable density formulation. Collagen fibers remodel through changes in radius and rest length, so the total collagen fiber volume can increase during the simulation. In the model, collagen remodeling changes the local fiber geometry, effective collagen density, stiffness, and anisotropic mechanical response without directly prescribing volumetric growth of the tissue domain, so the mass of collagen per unit total tissue volume can vary. Thus, tissue-level volume change is coupled primarily to the actomyosin/cellular component in the present model, while collagen contributes through changes in microstructure and mechanics. This is a modeling assumption, and alternative formulations, including collagen-driven volume expansion or constituent-specific mass balance laws, may also be appropriate in other growth and remodeling frameworks.

#### Discrete fiber networks

The fiber-scale remodeling framework is similar to that of Badal et al. (2025). In this formulation, each fiber evolves independently according to its local mechanical environment, following the principle of single-fiber tensional homeostasis (Flynn et al. 2010; Braeu et al. 2017; Walker et al. 2020; Stamenović and Smith 2020). The remodeling laws are defined on a fiber-by-fiber basis as

4 $$\frac{\hbox{d}{R}^{p}}{\hbox{d}t}=\frac{1}{\tau }\left(\frac{{P}^{p}}{{P}_{\infty }}-1\right){R}^{p}$$

5 $$\frac{\hbox{d}{L}^{p}}{\hbox{d}t}=\frac{1}{\tau }\left(\frac{{P}^{p}}{{P}_{\infty }}-1\right){L}^{p}$$

where ${R}^{p}$ and ${L}^{p}$ denote the instantaneous radius and length of fiber p, t is time, τ is the characteristic remodeling time constant, ${P}^{p}$ is the local axial first Piola–Kirchhoff stress, $\mathrm{a}\mathrm{n}\mathrm{d} \;{P}_{\infty }\;\mathrm{i}\mathrm{s}\;\mathrm{t}\mathrm{h}\mathrm{e}\;\mathrm{h}\mathrm{o}\mathrm{m}\mathrm{e}\mathrm{o}\mathrm{s}\mathrm{t}\mathrm{a}\mathrm{t}\mathrm{i}\mathrm{c}\;\mathrm{t}\mathrm{a}\mathrm{r}\mathrm{g}\mathrm{e}\mathrm{t}\;\mathrm{s}\mathrm{t}\mathrm{r}\mathrm{e}\mathrm{s}\mathrm{s}.\;{P}^{p}$ is equal to ${f}^{p}/{a}^{p}$, where ${f}^{p}$ is the fiber force, and ${a}^{p}$ is the undeformed cross-sectional area. When ${P}^{p}>{P}_{\infty }$, the fiber grows by increasing its radius and length; when ${P}^{p}<{P}_{\infty }$, it undergoes atrophy.

Actomyosin fibers remodel rapidly (*τ* = 4 days) toward a target stress of 0.75 MPa, reflecting their short turnover times and contractile adaptation (Steucke et al. 2017). Collagen fibers remodel over much longer timescales (*τ* = 90 days) toward a target stress of 2 MPa, consistent with gradual matrix deposition and cross-link maturation (Nissen et al. 1978; Mahutga and Barocas 2020). Elastin fibers are assumed to be permanent due to their long half-life (Shapiro et al. 1991) and therefore do not remodel, except to represent elastin fragmentation as described below, within the timescale of interest.

The current formulation assumes that no new fibers are created and no existing fibers are removed during the simulation period. Instead, remodeling occurs through local geometric adaptation of existing fibers, representing molecular-scale assembly or degradation. After each remodeling update, the modified network is reintroduced into the NaFNS solver (Mahutga et al. 2023), which recalculates the mechanical equilibrium for the new configuration. This iterative procedure enforces localized tensional homeostasis, driving each fiber toward its individual stress target and, collectively, bringing the tissue to a new equilibrium configuration consistent with the imposed deformation and boundary conditions. The explicit unloaded configuration of the DFN is not tracked explicitly; instead, remodeling occurs through incremental geometric updates to fiber length and radius, with equilibrium recalculated after each step.

#### Neo-Hookean component

Explicit calculation of the unloaded configuration of the neo-Hookean component is required and is effected as follows. Unlike earlier versions of our remodeling model (Gacek et al. 2022; Badal et al. 2025), in which fibers changed size to achieve homeostatic stress while the tissue experienced only remodeling at the macroscopic scale (equivalent to a variable constituent density model), the present formulation incorporates an explicit growth component into the bulk neo-Hookean matrix. This addition enables the model to capture geometric adaptations observed in the ascending aorta with Marfan syndrome. The growth tensor in this work depends directly on the evolution of the actomyosin network, which represents the collective contractile and adaptive behavior of cells within the tissue.

Since actomyosin fibers are modeled using the constant density approach, the space occupied by that element is determined by its mass. Thus, an increase or decrease in the mass of actomyosin fibers due to remodeling results in a corresponding change in the tissue volume. Within a growth framework, this volumetric change is represented by an inelastic growth deformation gradient for the neo-Hookean material, ${F}^{g, NH}$, which captures the size change of actomyosin fibers due to constituent deposition or removal. In soft tissues, such growth is commonly modeled as a volumetric process occurring at constant material density (Braeu et al. 2017; Grytsan et al. 2017; Paukner et al. 2024). Guided by physiological observations, we introduce the growth tensor as,

6 $${F}^{g, NH}={\left(\frac{{m}_{\mathrm{actin}}}{{M}_{\mathrm{actin}}}\right)}^{1/3}I$$

where ${m}_{\mathrm{actin}}$ and ${M}_{\mathrm{actin}}$ are the current and initial constituent mass, respectively, and I is the identity tensor. In the present model, the ratio ${m}_{\mathrm{actin}}/{M}_{\mathrm{actin}}$ is computed from the total actomyosin fiber volume, so the growth ratio reflects the change in cellular volume from the initial configuration. In this way, growth at the tissue scale emerges naturally from cell-level remodeling activity within the network. The tensor ${F}^{g, NH}$ defines the unloaded configuration of the neo-Hookean component of the remodeled tissue.

### Interpretation of DFN-driven growth and remodeling in a macroscopic context

**Fig. 2:**
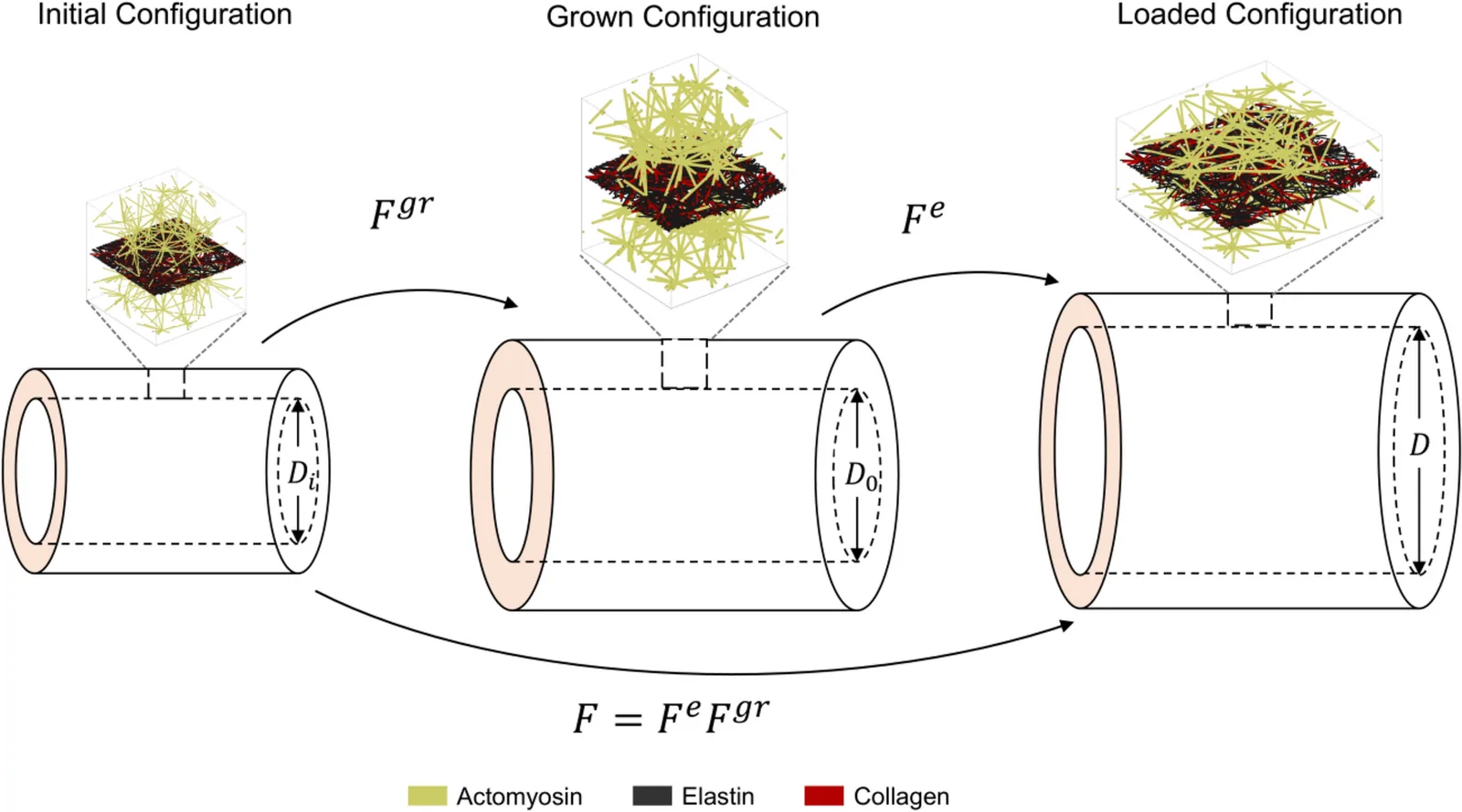
Schematic of the kinematic growth framework based decomposition of deformation, *F* = *F*^*e*^*F*^gr^, showing the initial unloaded configuration (*D*_*i*_), the grown unloaded configuration (*D*_0_), and the loaded configuration (*D*). Insets depict representative fiber-network configurations at each stage

The macroscale behavior of our model can be interpreted within the kinematic growth framework (Rodriguez et al. 1994), in which the deformation gradient F is multiplicatively decomposed into an inelastic contribution ${F}^{\mathrm{gr}}$ and an elastic contribution ${F}^{e}$ (Fig. 2). The tensor ${F}^{\mathrm{gr}}$ represents the inelastic changes in tissue geometry due to growth and remodeling, whereas ${F}^{e}$ captures the elastic deformation applied to the evolving configuration. In our formulation, the system begins from a fictitious reference state with initially isotropic fibers. The applied deformation F is imposed and held constant, while ${F}^{\mathrm{gr}}$ evolves in time to reflect growth and remodeling. Consequently, we write.

7 $$F={F}^{e}{F}^{\mathrm{gr}}$$

At t=0, when no growth and remodeling has occurred, ${F}^{\mathrm{gr}}=I$, and thus ${F}^{e}=F$. As remodeling proceeds, ${F}^{\mathrm{gr}}$ departs from the identity, and ${F}^{e}$ is updated accordingly. Conceptually, F reflects the applied deformation from the initial configuration, ${F}^{\mathrm{gr}}$ maps the initial to the evolving unloaded state, and ​ ${F}^{e}$ represents the elastic deformation from that evolving state. The constrained mixture model assumption implies that ${F}^{\mathrm{gr}}$ is the same for the fiber network and the bulk matrix and defines the unloaded configuration of the tissue as a whole. 

The inelastic part ${F}^{\mathrm{gr}}$ is often further decomposed into distinct contributions from growth and remodeling (Braeu et al. 2017):

8 $${F}^{\mathrm{gr}}={F}^{r}{F}^{g}$$

where ${F}^{g}$ is the growth tensor and ${F}^{r}$ is the remodeling tensor. In our case, since we impose only isotropic growth, ${F}^{g}$ is isotropic and ${F}^{r}$ is naturally isochoric (Gierig et al. 2024). In our modeling scheme, ${F}^{r}$ is perhaps a misnomer when called a “remodeling deformation gradient” since it is not driven directly by remodeling but is rather the correction to ${F}^{g}$ that arises because an anisotropic DFN interacts with the isotropic neo-Hookean growth to produce anisotropic tissue growth. ${F}^{r}$ changes as the fibers in the DFN remodel, but it represents DFN-matrix interaction, not remodeling per se. Here, ${F}^{g}={F}^{g, NH}$, and ${F}^{r}$ can be determined to get the unloaded geometry. These calculations are not needed for the computer model but are useful in post-processing to determine the unloaded tissue dimensions.

The distinct roles of the growth tensor ${F}^{g}$ and the remodeling tensor ${F}^{r}$ can be clarified by considering a set of simplified hypothetical cases applied to the layered microdomain used throughout this study. These idealized scenarios illustrate how volumetric change, shape change, and microstructural interactions emerge in our discrete fiber formulation, and why these behaviors differ from classical constrained mixture theory.

#### Scenario 1: collagen radius and length increase, but tissue volume remains unchanged (Fig. 3a)

**Fig. 3:**
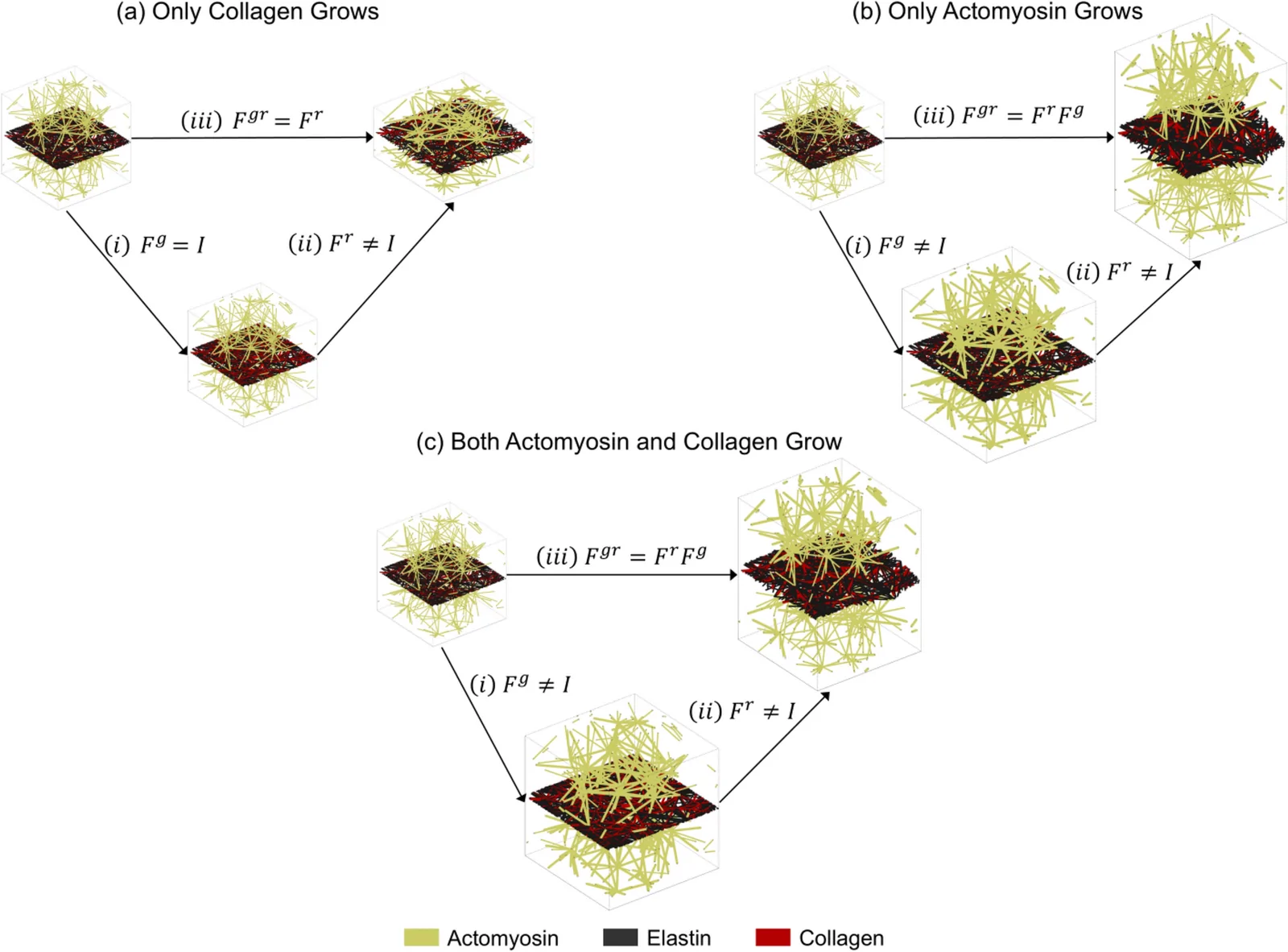
**a** When only collagen grows, there is no change in the unloaded volume (*F*^*g*^ = *I*, branch (i)), but the altered stiffness could lead to anisotropy, which would make *F*^*r*^ ≠ *I* (branch (ii)).** b** When only actomyosin grows, the unloaded volume changes (*F*^*g*^ ≠ *I*, branch (i)) and the fiber–fiber interaction would make *F*^*r*^ ≠ *I* (branch (ii)).** c** When actomyosin and collagen grow together, *F*^*g*^ ≠ *I* (branch (i)) due to change in unloaded volume and *F*^*r*^ ≠ *I* (branch (ii)) due to anisotropy

We consider first a case in which only collagen fibers increase in radius and rest length. Because collagen is modeled under the variable constituent density assumption, these geometric changes do not alter the overall volume of the microdomain. Thus, the growth tensor remains the identity, ${F}^{g}=I$ (branch (*i*)). However, collagen remodeling does induce internal shape change. As individual collagen fibers enlarge, their increased rest lengths and radii alter the forces exerted on adjacent elastin and actomyosin fibers through shared network nodes. At equilibrium, collagen fibers tend to be locally compressed, while actomyosin and elastin fibers carry slight compensatory tension. The microdomain changes shape by becoming longer within the collagen–elastin plane and shorter in the direction normal to that plane while still preserving volume. This anisotropic shape change is represented entirely through the remodeling tensor:

9 $${F}^{r}\ne I,{ F}^{g}=I,{ F}^{\mathrm{gr}}={F}^{r}$$

The effect is small because of the tension–compression asymmetry, but this scenario demonstrates that remodeling alone can generate tissue-level deformations even in the absence of volumetric growth.

#### Scenario 2: actomyosin volume increases and drives true volumetric growth (Fig. 3b)

Next, we consider a case in which actomyosin fibers increase their mass and volume, following constant constituent density. In this scenario, actomyosin enlargement necessarily increases the overall tissue volume, leading to a nontrivial growth tensor, ${F}^{g}\ne I$ (branch (i)). Actomyosin fibers grow longer and thicker, and because they are connected to elastin and collagen via nodes, their expansion induces both volumetric and shape changes. Since the collagen and elastin anisotropically resist the volume expansion, a nontrivial ${F}^{r}$ arises (branch (ii)). One might expect that if collagen maintains variable density, no additional geometric distortion would occur. However, the node-level coupling in the DFN means that the extending actomyosin fibers can put the elastin and collagen fibers into tension. The resulting equilibrium configuration is a domain that is larger overall due to volumetric growth, and anisotropically elongated. Thus, even when collagen contributes no direct volumetric growth, the mechanical consequences of actomyosin growth can still alter the remodeling tensor:

10 $${F}^{r}\ne I,{F}^{g}\ne I,{ F}^{\mathrm{gr}}={F}^{r}{F}^{g}$$

This behavior arises from both microstructural coupling and mixture–theory interaction between the DFN and the neo-Hookean solid, highlighting a fundamental difference between our approach and continuum-scale constrained mixture formulations.

#### Scenario 3: combined collagen and actomyosin-driven growth and remodeling (the case in this study, Fig. 3c)

In the full model used in this work, both constituents remodel. Because fibers interact mechanically through nodal connections, the geometric effects of actomyosin and collagen growth are not separable. Actomyosin-driven growth modifies the stress environment of collagen and elastin, while collagen remodeling shifts node positions in ways that feed back into actomyosin loading. Consequently, ${F}^{g}$ is governed exclusively by actomyosin volumetric change but ${F}^{r}$ depends on all constituents. Although actomyosin is added at constant density with no prescribed shape change, its presence within a tightly coupled network means that actomyosin expansion simultaneously produces tissue-level volume changes and alters the geometry of the other constituents. This is a central distinction between our framework and classical constrained mixture theory, in which constituents often act mechanically in parallel rather than through discrete junctions. In our model, microstructural coupling is explicit, and growth and remodeling emerge as collective network-driven phenomena.

### Elastin fragmentation

**Fig. 4:**
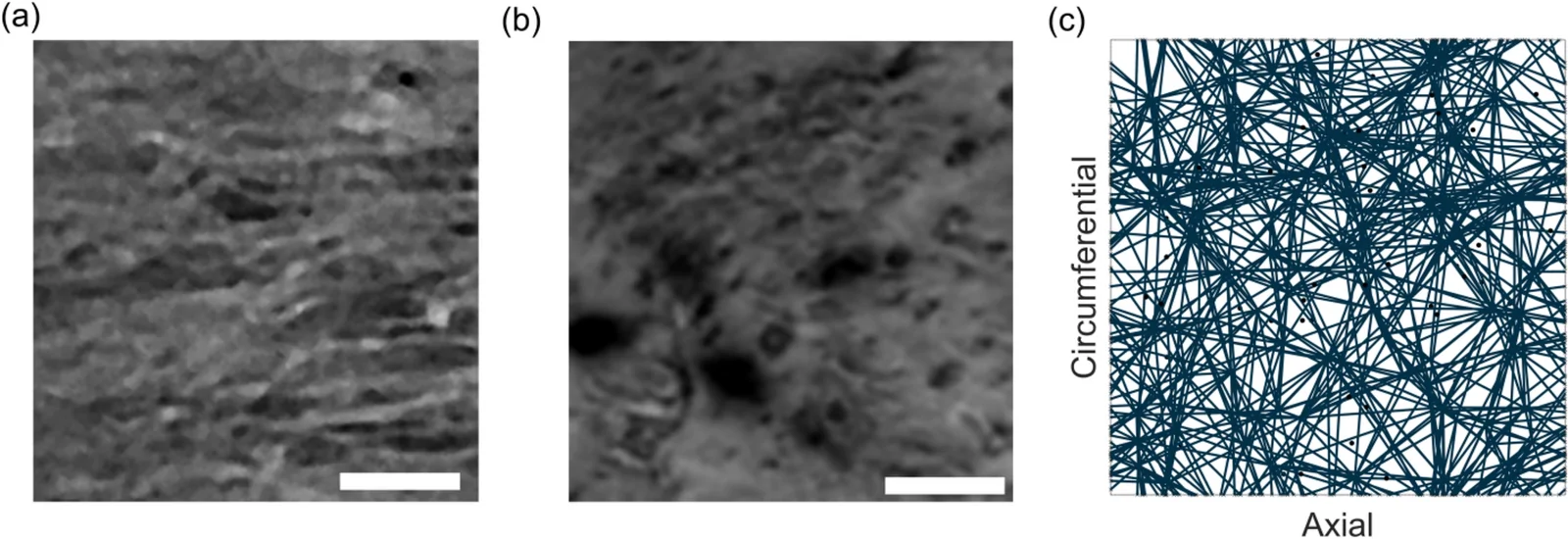
**a** Representative en face multiphoton images of the ascending aorta from wild-type mice and **b** Marfan mice, illustrating disrupted elastin architecture in Marfan vessels. Scale bar = 50 μm **c** Schematic representation of an elastin network. All fibers lose volume equally through radius reduction in the Marfan models

Elastic fiber fragmentation is a defining feature of Marfan-syndrome-associated aneurysms (Wanga et al. 2017). Despite the importance of the underlying biological mechanisms, our focus in this study was on mechanics and multiscale growth and remodeling of the vessel wall. Elastin loss was therefore implemented as a driver for remodeling rather than a downstream consequence of molecular regulation. Instead of modeling the chemical pathways leading to fragmentation explicitly, we impose elastin degradation directly within the fiber-scale representation of the tissue based on experimental observations. Experimental data from Dwivedi et al. (2024) indicated that elastin content, quantified as pixel intensity per area, decreased substantially in Marfan mice within the first three months and then remained approximately constant between months three and four (Fig. 4a and b). In this study, all elastin fibers underwent a uniform loss of radius over time (Fig. 4c). The DFN in Fig. 4 is a schematic representation of elastin-loss implementation and is not intended to reproduce the full wavy, multilamellar architecture of the aortic media. The radius of each elastin fiber in the model was linearly reduced during the first 90 days of remodeling. After this period, elastin geometry was held fixed while growth and remodeling of the remaining constituents proceeded to completion over 120 days.

### Implementation

In the present model, a finite element was initialized from a fictitious reference configuration with a prescribed base network and hypothetical radius and wall thickness. This configuration did not correspond to a physically realizable state but served as the undeformed reference for subsequent growth and remodeling. The tissue was then held under prescribed circumferential and axial stretches, during which growth and remodeling proceeded until equilibrium was reached. After equilibration, the element was released from the applied stretches and allowed to expand freely. The ratio of the relaxed element dimensions to their original reference values defined ${F}^{\mathrm{gr}}$. ${F}^{g}$ was calculated dynamically at each simulation step based on the evolving actomyosin volume, as described previously. ${F}^{e}$ was computed as the ratio of the initially applied macroscopic deformation to the combined effects of growth and remodeling (Eq. (7)). At each time step, ${F}^{e}$ was passed to the neo-Hookean material formulation to calculate the matrix stress, and the resulting circumferential stress, ${\sigma}_{\theta \theta }={\sigma}_{\theta \theta }^{\mathrm{DFN}}+{\sigma}_{\theta \theta }^{\mathrm{NH}}({F}^{e})$, was used to determine the intramural pressure according to

11 $$P=\frac{h}{R}\,{\sigma}_{\theta \theta }$$

where h and R denote the current deformed wall thickness and radius, respectively. The applied stretches and initial network configuration were iteratively adjusted until a physiological pressure close to 105 mmHg was achieved. At this pressure, the model reproduced the target elastic axial and circumferential stretches corresponding to the wild-type condition.

The hypothetical initial wall thickness and radius were then calculated as the current values divided by the radial and circumferential components of the total growth–remodeling deformation. This procedure established a baseline wild-type state consistent with experimental measurements of stresses, fiber contributions, and overall mechanical response. Low-pressure and high-pressure circumferential moduli were calculated from linear fits to the stress–stretch curves obtained under controlled circumferential stretching while axial stretch was held at the in vivo value. Stresses were converted to pressures using the law of Laplace. Following the approach of the experimental study (Dwivedi et al. 2025), the low-pressure modulus corresponded to pressures between 0 and 60 mmHg, and the high-pressure modulus to 120–150 mmHg, representing elastin- and collagen-dominated mechanical responses, respectively (Lammers et al. 2008; Dwivedi et al. 2023). The pressure ranges were chosen to match those in the experimental study of Dwivedi et al. (2025).

To investigate the influence of elastin degradation, the total elastin volume loss was varied from 10 to 80% in increments of 10%. For each level of elastin loss, geometric, mechanical, and microstructural metrics were computed. For each metric, a normalized error parameter was calculated as a *z* score,

12 $$e=\frac{\left|{X}_{S}-{X}_{E}\right|}{\mathrm{S}\mathrm{D}({X}_{E})}$$

where $X={\phi}_{\mathrm{M}\mathrm{S}}/{\phi}_{\mathrm{W}\mathrm{T}}$ is the ratio of the property ϕ for the Marfan syndrome and wild-type cases, the subscripts S and E refer to simulation and experiment, respectively, and SD is the standard deviation. This use of *z* score allowed quantitative comparison between simulation predictions and experimental observations across different levels of elastin loss and different measurements. All experimental data in this paper were taken from Dwivedi et al. (2025) (Table 2), for wild-type mice, *n* = 10 for all measurements except high-pressure modulus, *n* = 9; for Marfan mice, *n* = 5 for all measurements; only male mice were included, and all mice were 4 months old.

**Table 2:** Experimental results from Dwivedi et al. (2025)

Quantity	Wild-type	Marfan mice
Wall thickness [μm]	134 ± 9.5	167 ± 22
Vessel radius [μm]	447 ± 29	669.5 ± 216.5
Axial stretch	1.79 ± 0.26	1.48 ± 0.13
Axial stress [kPa]	265 ± 57	217 ± 128
Circumferential stretch	1.79 ± 0.21	1.82 ± 0.12
Circumferential stress [kPa]	267 ± 92	256 ± 98
Low-pressure modulus [kPa]	290 ± 70	162 ± 58
High-pressure modulus [kPa]	1084 ± 525	4915 ± 966

Values are mean ± SD (*n* = 5 to 10 (see text))

### Model limitations

The remodeling laws used here are phenomenological and do not explicitly resolve the cellular processes responsible for tissue turnover. Changes in collagen fiber radius and rest length represent the net effects of matrix deposition and degradation within the existing network. Similarly, actomyosin is a coarse-grained representation of the cellular and contractile smooth muscle component; changes in actomyosin content represent net cellular volume adaptation but do not explicitly resolve cell proliferation, hypertrophy, or intracellular regulation. The model represents biological growth through changes in existing fiber geometry and the coarse-grained cellular component. It does not explicitly simulate the creation or removal of complete collagen fibers, cell division, or cellular hypertrophy. These processes are represented only through their net mechanical and volumetric consequences.

These simplifications reflect a trade-off between biological and structural detail. Classical constrained mixture models explicitly represent constituent production, removal, survival, and deposition histories (Humphrey and Rajagopal 2002; Paukner et al. 2024), whereas the present DFN resolves local fiber topology, non-affine deformation, heterogeneous loading, and direct mechanical coupling among constituents (Mahutga and Barocas 2020; Gacek et al. 2022; Mahutga et al. 2023). The two approaches therefore address complementary aspects of growth and remodeling. Future work coupling cell-signaling and constituent-turnover models with the DFN could introduce additional biological detail while retaining its microstructural resolution.

Elastin degradation is prescribed directly rather than generated through FBN1-associated signaling, altered TGF-β regulation, MMP activity, or other biochemical pathways. The model therefore begins downstream of the biological processes responsible for elastin fragmentation and isolates the subsequent mechanical consequences of elastin loss.

### Statistics and data analytics

All statistical analyses were performed using Python (Python Software Foundation, Wilmington, DE, USA). Each simulation condition was evaluated using five independently generated discrete fiber networks with identical numbers of nodes to account for variability arising from random network generation. Computational results are therefore reported as the mean ± SD across these five network realizations, and error bars in all figures represent SD. For comparisons between each elastin-loss case and the base simulation, statistical significance was assessed using two-tailed Student’s *t*-tests. A significance level of p<0.05 was considered statistically significant.

### Target-stress sensitivity analysis

A sensitivity analysis was performed using Periodic Network 1 to evaluate the influence of the homeostatic target stresses. The base case and all elastin-loss conditions from 10 to 80% were rerun after increasing the actomyosin target stress by 10% and, separately, increasing the collagen target stress by 10%. For each perturbation, Marfan-to-base ratios were calculated for the same eight geometric and mechanical metrics used in the normalized error analysis. Similarity to the original simulations was quantified using the mean absolute percent difference between the sensitivity and original ratios across the 10–80% elastin-loss cases; the 0% case was excluded because its Marfan-to-base ratio is unity by definition. The average normalized error was recalculated at each elastin-loss level, and a quadratic fit was used to determine the elastin-loss level corresponding to the minimum error.

## Results

### Pressure normalization and baseline calibration

**Fig. 5:**
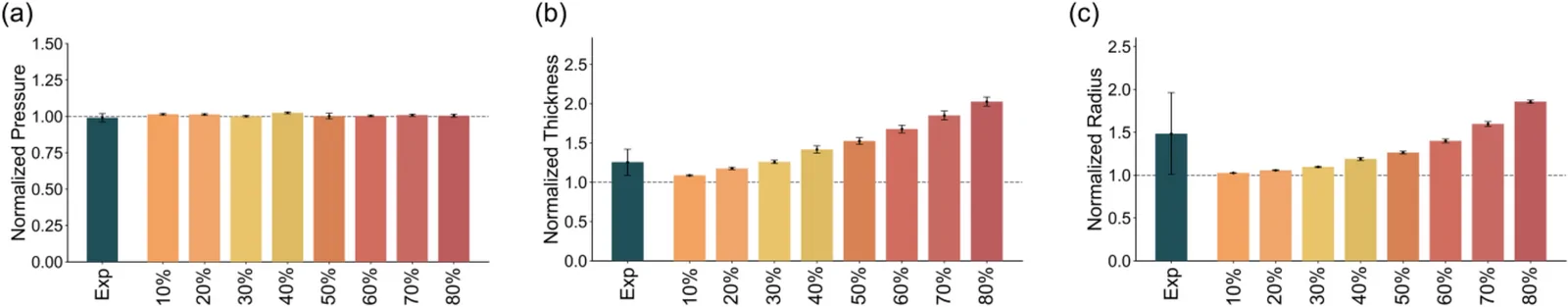
Experimentally measured ratio between wild-type and Marfan mice alongside computational predictions for the elastin-loss levels. **a** Pressure ratio is close to unity for all cases. **b** Marfan aortas are generally thicker (ratio > 1). **c** Marfan aortas tend to have larger radii (ratio > 1)

Experimental measurements showed no significant difference in mean arterial pressure between wild-type and Marfan mice. To ensure consistency across simulations, each computational case was independently remodeled to a target intramural pressure of approximately 105 mmHg. The resulting pressures at equilibrium were tightly clustered across all simulations (Fig. 5a), confirming successful normalization of the loading condition. The base simulation was calibrated to match the experimentally measured wild-type ascending aortic geometry. From this configuration, the hypothetical unloaded wall thickness ${t}_{i}$ and radius ${R}_{i}$ were computed for each replication, yielding mean values of ${t}_{i}=129.1\, \upmu \hbox{m}$ and ${R}_{i}=441.3\, \upmu \hbox{m}$. For each elastin-loss level, the unloaded dimensions were then mapped to the ex vivo configuration via the radial and circumferential components of the total growth–remodeling deformation, such that

13 $${t}_{0}={t}_{i}{\lambda}_{r}^{\mathrm{gr}},{R}_{0}={R}_{i}{\lambda}_{\theta }^{\mathrm{gr}}$$

### Wall thickness and radius

Experimentally, the ex vivo (i.e., unloaded) wall thickness and radius of the ascending aorta were significantly larger in Marfan mice than in wild-type controls. In the computational framework, all elastin-loss levels produced increases in both wall thickness and radius relative to the base simulation (Fig. 5b and c). These increases were observed consistently across individual replications and case-averaged values. The magnitude of geometric enlargement increased monotonically with increasing elastin loss. Comparison with experimental measurements showed the smallest normalized error for wall thickness at approximately 30% elastin loss and for radius at approximately 60% elastin loss.

### Axial and circumferential mechanics

**Fig. 6:**
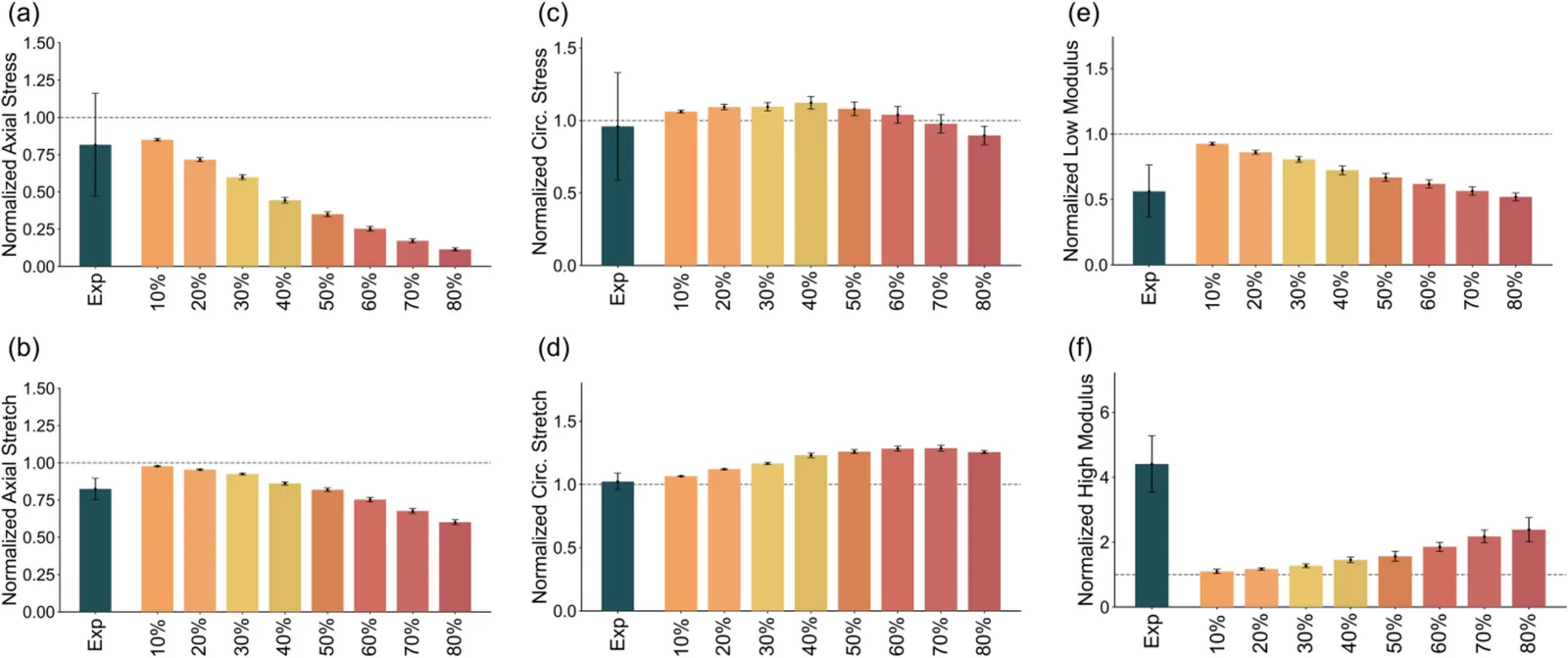
Experimental Marfan-to–wild-type ratios alongside computational predictions for the elastin loss levels. **a** Axial stress and **b** axial stretch are generally reduced in Marfan aortas compared to wild-type. **c** Circumferential stress remains largely unchanged experimentally, whereas simulations show loss-level dependent behavior. **d** Circumferential stretch exhibits minimal experimental change but a modest increase in all computational cases. **e** The low-pressure (elastin-dominated) modulus decreases in Marfan aortas, while **f** the high-pressure (collagendominated) modulus increases

Experimentally, the ex vivo estimates of in vivo axial stress decreased modestly from wild-type to Marfan mice. The computational model predicted a statistically significant reduction in in vivo axial stress across all elastin-loss levels, with values decreasing from approximately 280 kPa in the base simulation to between 220 and 30 kPa in the pathological simulations (Fig. 6a). Among the simulated cases, the 10% elastin-loss condition produced the closest agreement with experimental measurements. Both experimental and computational datasets showed a reduction in in vivo axial stretch from wild-type to Marfan conditions (Fig. 6b). Among the computational cases, the 50% elastin-loss condition yielded the smallest error relative to experimental measurements.In the circumferential direction, experimental measurements indicated that circumferential stress and stretch remained approximately unchanged between wild-type and Marfan mice. In contrast, computational results showed elastin-loss-dependent behavior. Circumferential stress increased initially with increasing elastin loss, reached a maximum at approximately 40% loss, and then decreased at higher loss levels, with some cases becoming statistically indistinguishable from the base simulation (60%, 70%). Although circumferential stress varied with elastin loss, the quadratic trend indicated only moderate deviation from the baseline value, suggesting partial preservation of circumferential stress. Circumferential stretch exhibited a small but statistically significant increase relative to the base simulation for all elastin-loss cases. Both circumferential stress and stretch therefore displayed non-monotonic behavior, with peak values occurring between approximately 40% and 70% elastin loss.

### Circumferential stiffness

Experimentally, Marfan mice exhibited a reduced low-pressure (elastin-dominated) circumferential modulus and an increased high-pressure (collagen-dominated) modulus relative to wild-type controls. The computational results reproduced these qualitative trends across all elastin-loss simulations: the low-pressure modulus decreased relative to the base simulation, whereas the high-pressure modulus increased (Fig. 6e and f). Quantitatively, the simulated low-pressure modulus values were lower than the corresponding experimental measurements, while the high-pressure modulus values were higher. The magnitude of both changes increased monotonically with increasing elastin loss. The lowest normalized error occurred at approximately 70% elastin loss for the low-pressure modulus and 80% elastin loss for the high-pressure modulus.

### Changes in fiber content

Experimental imaging revealed significantly increased elastin porosity in the ascending aorta with reduced elastin content (see Fig. 4b). In the computational framework, elastin loss was imposed directly according to each fragmentation scheme, while changes in collagen and actomyosin content emerged from growth and remodeling.

**Fig. 7:**
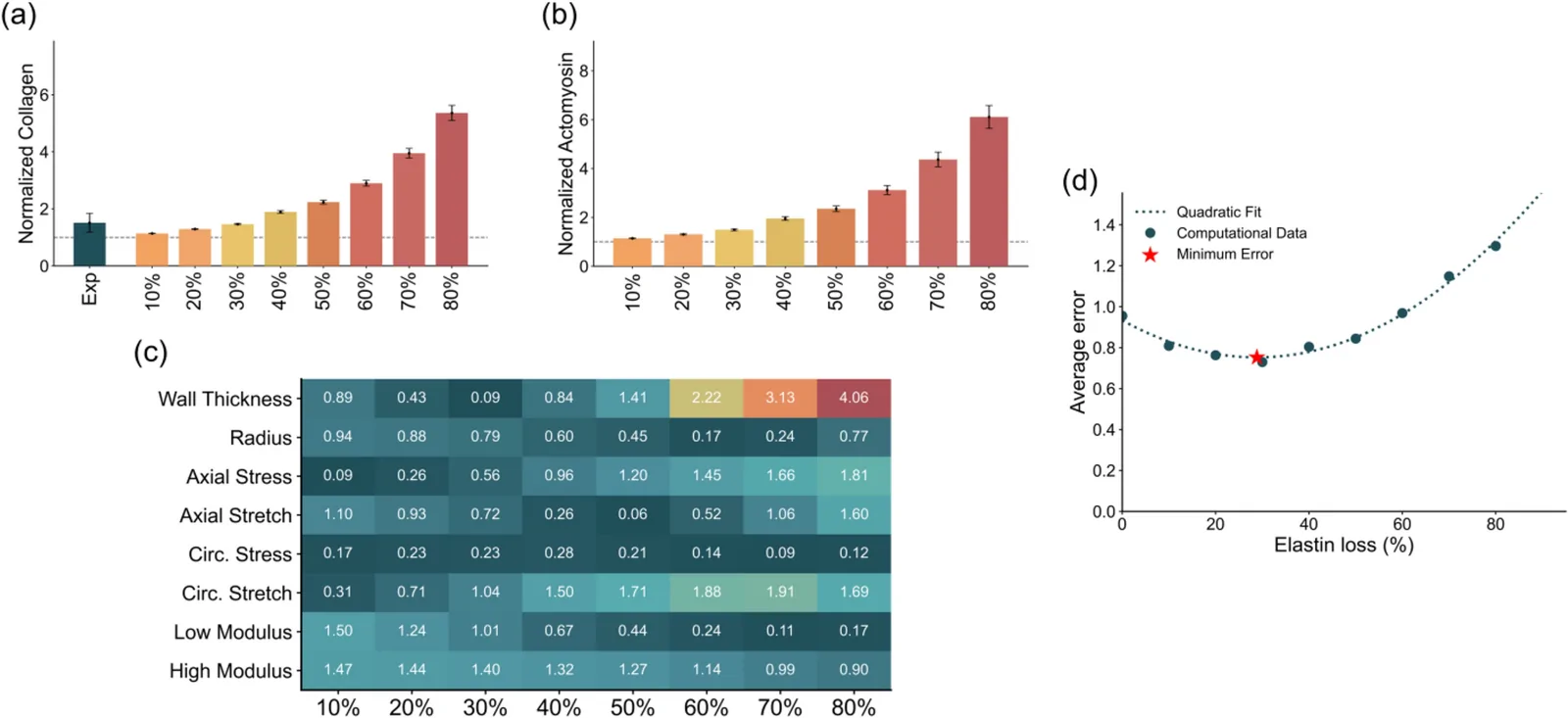
**a** Experimentally measured ratio of collagen content between wild-type and Marfan mice, alongside computational predictions show an increase in collagen volume. **b** Actomyosin volume increases in all elastindegradation schemes (ratio > 1). **c** Normalized errors heatmap for the eight predicted geometrical and mechanical parameters across elastin-loss levels **d** Average normalized error across eight geometric and mechanical metrics as a function of elastin loss. The error follows a quadratic trend (*R*^2 ^> 0.98) with a minimum occurring at approximately 29% elastin loss

Experimentally, collagen content was quantified using second harmonic generation method as intensity normalized by pixel area (intensity/pixel^2^). Using this metric, collagen proportion was significantly increased in the experimental measurements. In the computational framework, fiber abundance was quantified using total fiber volumes. All elastin loss levels yielded collagen and actomyosin ratios greater than one relative to the base simulation, indicating increased constituent content (Fig. 7a and b). The collagen increase in pathological simulations was consistent with the experimentally observed elevation in collagen proportion. The increase in actin and collagen volume was monotonic with higher elastin loss cases showing an increased volume gain. In addition to total collagen volume, the average collagen fiber radius increased in all pathological simulations.

### Overall performance

Figure 7c shows a heatmap of normalized errors for the predicted parameters (rows) across elastin-loss levels (columns). Because actomyosin content was not measured experimentally and collagen content was reported using a different metric than the computational model, these quantities were excluded from the error analysis. The normalized error from Eq. (12), averaged across the remaining eight measured parameters, was then computed for each elastin-loss level. Plotting this average error as a function of elastin loss revealed a quadratic relationship. Figure 7d shows the quadratic fit together with the discrete simulation results (*R*^2^>0.98). The minimum of this curve, indicated by a star in the plot, corresponds to an elastin-loss level of approximately 29%, representing the best agreement between computational predictions and experimental observations.

### Sensitivity to target stresses

**Fig. 8:**
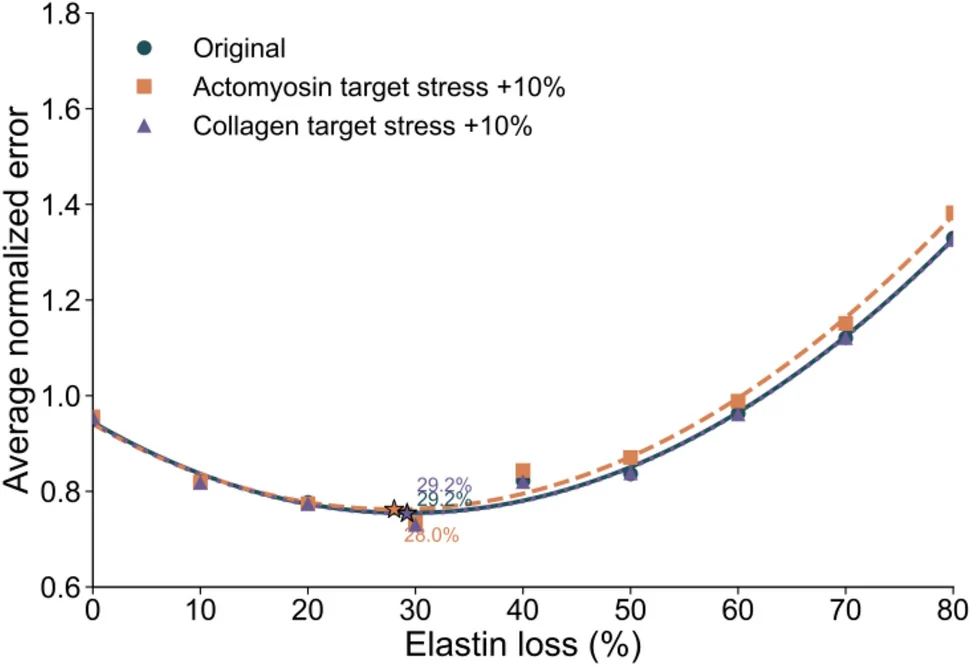
Sensitivity of the average normalized error to 10% increases in the actomyosin and collagen homeostatic target stresses for Periodic Network 1

Increasing the actomyosin target stress by 10% altered the absolute stresses, stretches, moduli, and geometric predictions, whereas increasing the collagen target stress produced comparatively smaller changes. However, the Marfan-to-base ratios remained similar to the original results. The mean absolute percent difference across the eight comparison metrics and 10–80% elastin-loss levels was 1.21% for the actomyosin perturbation and 0.67% for the collagen perturbation. The fitted minimum shifted from 29.23% elastin loss in the original case to 28.02% following the actomyosin target-stress increase and 29.21% following the collagen target-stress increase (Fig. 8). The corresponding minimum average normalized errors were 0.7548, 0.7625, and 0.7537, respectively. Thus, although the absolute model outputs were affected by the target stresses, the normalized trends and the predicted elastin-loss level producing the best overall agreement were relatively insensitive to these 10% parameter perturbations.

## Discussion

The central question of this study was whether elastin fragmentation, once present, could mechanically reproduce the geometric and functional abnormalities observed in the Marfan ascending aorta under the assumption of our growth and remodeling model. The present model does not establish a mechanistic pathway from FBN1 mutation to matrix remodeling. Instead, it tests whether elastin loss, once present, can mechanically generate key geometric and functional features of the Marfan aorta through fiber-scale remodeling. Specifically, we explored whether progressive loss of elastin redistributes mechanical load to the remaining structural constituents—primarily collagen and actomyosin—thereby triggering compensatory growth and remodeling. Under homeostatic regulation, each fiber family seeks to maintain its preferred stress state; consequently, increased load is accommodated through increases in fiber length and radius. Within our growth and remodeling framework, this adaptive response manifests as increased collagen and actomyosin content with the latter driving volume expansion, providing an explanation for the experimentally observed increases in wall thickness, radius, and stiffness in Marfan aortas relative to healthy controls.

In the model, the ex vivo wall thickness and radius arise from the combined effects of initial geometry and growth stretches in the circumferential and radial directions. As elastin is removed through degradation, the resulting load transfer leads to increased collagen and actomyosin production. Because the formulation enforces a constant cell density, with actomyosin representing the smooth muscle component, any increase in actomyosin content necessarily produces an isotropic increase in the growth tensor. Since the remodeling tensor is isochoric ($\mathrm{d}\mathrm{e}\mathrm{t}\,{ F}^{r}=1$), increases in the growth tensor directly amplify the total growth–remodeling deformation, yielding larger wall thicknesses and radii. From an experimental standpoint, this mechanism is fully consistent with reported structural changes in Marfan aortas and provides a possible explanation linking microstructural elastin loss to macroscopic vascular remodeling.

The model also provides an explanation for the reduced axial stretch observed experimentally in Marfan mice (Cavinato et al. 2021; Tarraf et al. 2024). We have previously argued that the axial length is externally imposed on a vessel by surrounding anatomy, and the axial stretch emerges as a consequence of growth and remodeling required to accommodate that imposed length (Badal and Barocas 2025). Both wild-type and Marfan vessels develop under the same externally constrained axial length of the ascending aorta. During postnatal remodeling, wild-type vessels require relatively little growth to maintain homeostasis, resulting in a higher in vivo axial stretch. In Marfan vessels, however, progressive elastin loss alters wall composition and load sharing, driving increased axial growth. This increases the unloaded axial length of the vessel, and because the in vivo length remains fixed, the ratio of loaded to unloaded length, and thus the in vivo axial stretch necessarily decreases. The reduced axial stretch in Marfan mice, therefore, reflects a changed unloaded configuration rather than a loss of axial constraint. Consistent with this interpretation, Dwivedi et al. (2026) recently reported an increased aortic elongation ratio, defined as the actual aortic centerline length divided by the geometric length, in severe Marfan mice relative to wild-type controls. Although this whole-aorta metric does not directly measure the unloaded length of the ascending aorta, it provides independent evidence of increased macroscopic aortic elongation.

The largely preserved circumferential stress observed in both the experimental dataset and the simulations provides additional support for the hypothesis of circumferential stress homeostasis. The uniform circumferential stress hypothesis (Kassab 2006) proposes that arteries remodel in order to maintain a relatively constant circumferential wall stress despite changes in geometry or composition. This concept aligns with the broader principle of tensional homeostasis at multiple length scales (Flynn et al. 2013; Stamenović and Smith 2020). Consistent with this framework, recent work by Badal et al. (2026) demonstrated that circumferential stress remains approximately uniform along the length of the healthy aorta. The minimal change in circumferential stress observed experimentally, together with its partial preservation in the simulations despite the absence of a prescribed tissue-level circumferential stress target, is consistent with the idea that fiber-scale homeostatic remodeling contributes to the maintenance of circumferential stress during elastin loss. This agreement should be interpreted as a model prediction rather than proof of the underlying in vivo regulatory mechanism.

Discrepancies between experimental and computational results in other mechanical quantities, particularly circumferential stretch, warrant consideration. These differences likely arise from a combination of modeling assumptions and experimental limitations. Importantly, the experimentally reported values are derived from ex vivo measurements and reconstructed stress–stretch calculations rather than direct in vivo observations. As a result, some quantitative disagreement is expected and does not undermine the qualitative agreement or the underlying mechanistic interpretation.

The pressure-dependent stiffness trends further support the proposed load-transfer mechanism. Elastin is generally accepted as the dominant load-bearing constituent at low pressures, whereas collagen governs mechanical response at higher pressures (Dobrin et al. 1984; Weisbecker et al. 2013). Loss of elastin therefore naturally leads to a reduction in low-pressure modulus, as observed experimentally in Marfan aortas and reproduced in the computational model. Simultaneously, the compensatory increase in collagen content explains the increase in high-pressure modulus seen in both experimental and simulated results. Together, these findings reinforce the interpretation that elastin fragmentation initiates a cascade of load redistribution and matrix remodeling that reshapes the mechanical behavior of the vessel across the pressure range.

In the present study, elastin degradation was represented as a uniform reduction in elastin fiber radius. While this approach captures the overall loss of elastin content observed experimentally, it represents only one possible mechanism of elastin fragmentation. Because the discrete fiber network explicitly resolves microstructural heterogeneity, the model also permits implementation of alternative degradation mechanisms, including localized pore formation, random fiber deletion, or mechanically driven failure of individual fibers. Exploring how such heterogeneous degradation patterns influence growth and remodeling responses would be a natural extension of the framework developed here. It is possible that for different degradation mechanisms, the best-fit elastin loss level and error distribution across metrics could change substantially.

The average normalized error across the eight geometric and mechanical metrics remained below one for elastin-loss levels up to approximately 60%, with a minimum occurring near 29% elastin loss. Because the error metric was defined relative to the experimental standard deviation, this result indicates that, on average, the model predictions for these quantities fell within one standard deviation of the corresponding experimental measurements. The minimum average error was approximately 0.75, suggesting that the computational framework can reproduce the overall mechanical and geometric phenotype of the Marfan ascending aorta with reasonable quantitative accuracy. The elastin-loss level associated with the minimum model error (~ 29%) lies within the qualitative range of elastin depletion observed experimentally in Marfan aortas. Because the experimental measurements quantify elastin intensity rather than volumetric fiber content, a direct quantitative comparison is not possible. Nevertheless, the consistency in magnitude supports the hypothesis that elastin degradation plays a major role in driving the observed remodeling response.

The present results should be interpreted in the context of prior constrained mixture modeling of Marfan-associated aortic remodeling. Li et al. (2023) concluded that elastin loss alone was not sufficient to reproduce the observed Marfan phenotype, whereas the present model predicts that prescribed elastin loss can reproduce several key geometric and mechanical changes. This difference likely arises from the different treatments of microstructural coupling. In continuum constrained mixture models, constituents generally contribute to the total stress through homogenized constituent-level responses. In the present DFN framework, collagen, elastin, and actomyosin are mechanically coupled through shared nodes, so degradation or remodeling of one constituent can directly alter the local mechanical environment of the others. Thus, elastin loss can redistribute load at the fiber scale, drive compensatory collagen and actomyosin remodeling, and produce tissue-level geometric changes. This distinction suggests that explicit microstructural coupling may be important for capturing how elastin fragmentation translates into medial growth and remodeling.

A limitation of the present study is that the model represents only the medial layer and does not explicitly include the adventitia. This choice was made to focus on the mechanical consequences of medial elastin fragmentation, which is a defining feature of Marfan-associated aortic remodeling, and to retain a computationally efficient single-element framework. However, the adventitia contributes to arterial mechanical stability, load bearing, and remodeling, particularly in aneurysmal disease. Omitting the adventitia may therefore influence the quantitative stress redistribution predicted by the model.

Beyond evaluating the central hypothesis, the present study establishes a computational framework for investigating Marfan-associated aortic remodeling from a multiscale growth and remodeling perspective. The analysis here focused on the severe Marfan mouse model (*Fbn1*^mgR*/*mgR^) characterized experimentally by Dwivedi et al. (2025), which provided detailed measurements of geometry and mechanics for calibration and validation. However, additional Marfan mouse models, exhibiting different severities of disease progression, have been reported, including *Fbn1*^C1041G*/*+^ (Cavinato et al. 2021) and mg^*Δ*lpn^ (Tarraf et al. 2024). The framework developed here can readily be extended to these models to investigate how differences in genetic mutation and disease severity influence remodeling trajectories.

## Supplementary Information

Below is the link to the electronic supplementary material.

Supplementary file1 (DOCX 29696 kb)

## Data Availability

All data supporting the findings of this study are available within the paper and its Supplementary Information.
