# Supplementary material for: Computational multiscale analysis of growth and remodeling in the ascending aorta of the Marfan mouse
Source: Biomech Model Mechanobiol. 2026 Oct 7;25(5):117. doi: 10.1007/s10237-026-02134-x (PMC13645934; doi:10.1007/s10237-026-02134-x)
Supplement: Supplementary file 1 [file 10237_2026_2134_MOESM1_ESM.docx]

**Detailed Methods for Discrete-Fiber-Network-Based Multiscale Model of Arterial Wall**

**S1. Purpose**

Our group has published multiple papers based on a multiscale scheme coupling a discrete-fiber network (DFN) model of a piece of arterial media with a continuum-level finite-element model of the vessel as a whole. This model can also include a separate model of the adventitia. It would be wasteful to write up a detailed description of the modeling methods in new papers when the work has already been published elsewhere, but we recognize that it is inconvenient and frustrating for readers to have to dig through multiple papers to get enough detail on the current work to appreciate it fully and/or replicate it. We have therefore written these detailed methods as a reference document for new work that uses the previously described methods.

**S2. Fiber Creation**

The discrete fiber networks are created following our previously established methodology (Gacek et al. 2023; Mahutga et al. 2023, 2025). In brief, nodes are placed at random within a cubic domain (representative volume element, RVE) utilizing MATLAB’s *rand()* function. These nodes are then repeated into a 3 × 3 × 3 grid where the original nodes are kept in the center of the grid. A Delaunay tessellation is performed on the full 3 × 3 × 3 grid, and the extraction of central block of nodes and fibers ensures a periodic network structure. From that base, networks with decreasing connectivity are generated if needed via controlled decimation of the original Delaunay networks, while maintaining the periodicity of the whole structure. An example of this construction procedure and the produced single network is exhibited in Figure S1(a). This procedure is selected for its efficacy in creating homogeneous networks that approximate the interconnected characteristics of soft-tissue microstructures. Furthermore, Delaunay networks provide a high degree of connectivity and thus mechanical stability. The initial 3D Delaunay networks can be flattened to create highly connected 2D planar layers, or a similar Delaunay tessellation can be performed in 2D to create lower-connectivity planar layers. The flattened networks can be used for 2D simulations or, more commonly in our studies of arterial wall, can be combined with 3D networks to produce complex microstructures (e.g., the medial lamellar unit in Figure S1(b)).

Figure S1 (a) Single fiber-type network; (b) Composite network that represents the medial lamellar unit.

For models of the arterial media, we create a medial lamellar unit typically in a roughly 10 μm × 10 μm × 10 μm space, as shown in Figure S1(b), containing three fiber families–actomyosin, collagen, and elastin. We create actomyosin network as a fully 3D isotropic Delaunay network. Elastin is produced as a 3D Delaunay network, which is subsequently flattened into the circumferential–axial plane to represent sheet-like lamellae with enhanced connectivity. Collagen is generated by performing a 2D Delaunay tessellation to the decimated elastin nodes and subsequently reducing connectivity to emulate longer, more sparsely cross-linked collagen fibers. All networks are considered as pin-jointed structures, with physical links introduced at intersections where actin fibers meet the elastin/collagen lamina. The resulting meso-scale structure comprises a highly cross-linked, planar elastin sheet, a mechanically coupled collagen network with reduced connectivity, and a surrounding 3D actomyosin cytoskeleton, altogether representing the important structural features of the medial lamellar unit. The physical size of the RVE can be obtained using a scaling factor, denoted by $\chi$, following Refs. [4,5] and defined as

$\chi=\sqrt{\frac{\sum l_{f}A_{f}}{\phi V}}$	$\left( 1 \right)$

where$A_{f}$ denotes the cross-sectional area of fiber $f$, $l_{f}$ denotes the length of fiber $f$ in computational units, $V$ indicates the RVE volume in computational units cubed, and $\phi$ represents the fiber volume fraction. Although $V$ and $l_{f}$are defined in computational units, $A_{f}$ is specified in physical units, resulting in $\chi$ having dimensions of physical length per unit computational length. In the simulations, $\chi$ is treated as a constant and serves solely to map computational length scales of the RVE and fibers to their corresponding physical dimensions. By employing this scaling parameter, the volume-averaged stress within the RVE is attained by summing the forces contributed by all fibers.

**S3. Individual Network Mechanics**

We employ multiple fiber model types, leveraging the fact that fibers can be fully characterized by specifying a 1D force–stretch relationship. The three fiber systems in the medial lamellar unit–collagen, elastin, and actomyosin–are each assigned distinct constitutive models to capture their biological and mechanical roles:

***Collagen***

Collagen fibers are represented using a nonlinear helical spring formulation following the work of Freed and Doehring (Freed and Doehring 2005). We obtain the material parameters based on Flynn et al. (Flynn et al. 2010). The helical model accounts for three geometric parameters: a helix height ($H_{0}$), a fibril radius ($r_{0}$), and a helix radius ($R_{0}$), as indicated in Figure S2, which collectively creates a characteristic force–stretch curve that is initially compliant but rapidly stiffens as the helix uncoils (Flynn et al. 2010). The fiber’s chord length ($L_{0}$) can be expressed as $L_{0}= \sqrt{\left( 2\pi R_{0} \right)^{2}+H_{0}^{2}}$. Straightening of the helical structure occurs at a critical stretch ($\bar{\lambda}$), which is defined as $\bar{\lambda}=\frac{L_{0}}{H_{0}}$.

For a given fiber stretch ($\lambda^{e}$), the helix height (H) can be computed as $H=\lambda^{e}H_{0}$, while the corresponding helix radius (R) can be obtained using $R=\frac{\sqrt{L_{0}^{2}-H^{2}}}{2\pi}$ expression. At the transition between the heel and linear regions, the secant modulus ($\bar{E}$) is expressed by

$\begin{aligned} \bar{E}=\frac{EH_{0}^{2}}{L_{0}\left[ H_{0} + \left( 1 + \frac{37}{6\pi^{2}} + \frac{2L_{0}^{2}}{\left( \pi r_{0} \right)^{2}} \right)\left( L_{0}- H_{0} \right) \right]} \#\left( 2 \right) \end{aligned}$

where $E$ is the elastic modulus of the linear region. Moreover, the fiber axial force ($f^{e}$) in collagen fibers is expressed as

$\begin{aligned} f^{e}=\left\{ \begin{matrix} \bar{E}A^{e}\left( \lambda^{e}-1 \right) + EA^{e}\left( \frac{\lambda^{e}}{\bar{\lambda}}-1 \right) \text{ }\mathrm{if}\text{ }\lambda^{e}>\bar{\lambda} \\ \bar{E}A^{e}\xi\left( \lambda^{e}-1 \right) \text{ }\mathrm{otherwise} \end{matrix} \right.\#\left( 3a \right) \end{aligned}$

where $A^{e}$ is the cross-sectional area, and $\xi$ is a geometric parameter which is given by

$\begin{aligned} \xi=\frac{R^{2}+H^{2}}{L_{0}H\left[ 1+\frac{4R^{2}}{r_{0}^{2}}+6\left( \frac{20}{9}+\frac{R^{2}}{r_{0}^{2}} \right)\frac{R^{2}}{H^{2}} \right]} \#\left( 3b \right) \end{aligned}$

The fiber stiffness $(\frac{ⅆf^{ⅇ}}{ⅆ\lambda^{e}})$can be derived easily from eqn. (2) as $f^{e}$depends on fiber stretch with static geometric parameters.

***Elastin***

We represent elastin fibers as tension–compression asymmetric linear elastic elements, consistent with their propensity to buckle under compression due to high aspect ratio (Aaron and Gosline 1981). The constitutive relation for elastin fibers is defined as

$\begin{aligned} f^{e}=\left\{ \begin{aligned} EA^{e}\left( \lambda^{e}-1 \right) \lambda^{e}\geq1 \\ \frac{EA^{e}}{100}\left( \lambda^{e}-1 \right) \lambda^{e}<1 \end{aligned} \right.\#\left( 4 \right) \end{aligned}$

where $f^{e}$ is the fiber force, $\lambda^{e}$ is the fiber stretch, $E$ is the fiber modulus, and $A^{e}$is the undeformed cross-sectional area. The bilinear relation captures the near-linear tensile response and the drastic declination in load bearing under compression due to buckling (Mahutga et al. 2023). The stiffness is given by

$\begin{aligned} \frac{ⅆf^{ⅇ}}{ⅆ\lambda^{e}}=\left\{ \begin{aligned} EA^{e} \lambda^{e}\geq1 \\ \frac{EA^{e}}{100} \lambda^{e}<1 \end{aligned} \right.\#\left( 5 \right) \end{aligned}$

Figure S2 Geometrical parameters for the helical model used for collagen.

***Actomyosin***

Actomyosin fibers are represented as contractile structures having both active and passive components (Rachev and Hayashi 1999). The total fiber force ${(f}^{e})$is expressed as

$f^{e}=f^{p}+f^{a}$	$\left( 6 \right)$

where the passive component $(f^{p})$ follows the same bilinear relation as elastin, which is much more compliant in compression and given by

$f^{p}=\left\{ \begin{aligned} EA^{e}\left( \lambda^{e}-1 \right) \lambda^{e}\geq1 \\ \frac{EA^{e}}{100}\left( \lambda^{e}-1 \right) \lambda^{e}<1 \end{aligned} \right. \left( 7 \right)$

However, the active component $(f^{a})$is stretch-dependent and given by the following equation:

$f^{a}=\left\{ \begin{aligned} S_{0}A^{a}[1-{(\frac{\lambda^{\max}-\lambda^{e}}{\lambda^{\max}-\lambda^{\min}})}^{2}] \lambda^{\min}\leq\lambda^{e}\leq2\lambda^{\max}-\lambda^{\min} \\ 0 \mathrm{otherwise} \end{aligned} \right. \left( 8 \right)$

Here, $\lambda^{\max}$ is the stretch at maximum contractility, $\lambda^{\min}$ is the stretch at minimum contractility, $A^{a}$ is the undeformed fiber cross-sectional area, and $S_{0}$ is the maximum active contractile stress occurring at ${\lambda=\lambda}^{\max}$. The contractile stress is zero at ${\lambda=\lambda}^{\min}$ and at $\lambda=2\lambda^{\max}-\lambda^{\min}$, defining the bounds of active force generation. This representation showcases the dual role of stress fibers as passive load-bearing elements, as well as active force generators within the cytoskeleton. The stiffness is given by

$\frac{df^{e}}{d\lambda}=\frac{df^{a}}{d\lambda}+\frac{df^{p}}{d\lambda}=\left\{ \begin{aligned} EA^{e} \lambda^{e}>2\lambda^{max}-\lambda^{min} \\ 2S_{0}A^{e}\frac{\lambda^{max}-\lambda}{\left( \lambda^{max} - \lambda^{min} \right)^{2}}+EA^{e} 1\leq\lambda^{e}\leq2\lambda^{max}-\lambda^{min} \\ 2S_{0}A^{e}\frac{\lambda^{max}-\lambda}{\left( \lambda^{max} - \lambda^{min} \right)^{2}}+\frac{EA^{e}}{100} \lambda^{min}\leq\lambda^{e}<1 \\ \frac{EA^{e}}{100}\lambda^{e}<\lambda^{min} \end{aligned} \right.$ $\left( 9 \right)$

Material parameters for all three fiber types are adapted from experimental literature and are summarized in Table S1. Using these material parameters, the corresponding 1D stress–stretch relationships for collagen, elastin, and actomyosin fibers are shown in Figure S3. It should be noted that the specific values of the material parameters listed in Table S1 may vary across studies; consequently, the quantitative stress–stretch responses shown in Figure S3 may also differ from

Table S1 Representative material parameters of three fibers. It should be noted that the material parameters listed here may vary across studies.

| **Fiber Type** | **Material Parameters** | **References** |
| --- | --- | --- |
| Collagen | $H_{0}$ = 67.4 nm; $R_{0}$ = 5.8 nm; $r_{0}$ = 1.6 nm; $E$ = 700 MPa | (Aaron and Gosline 1981; Gosline et al. 2002) |
| Elastin | $E$ = 2 MPa | (Rachev and Hayashi 1999) |
| Actomyosin | $\lambda^{\max}$ = 1.4; $\lambda^{\min}$ = 0.65; $S_{0}$ = 0.2 MPa; $E$ = 1.6 MPa | (Freed and Doehring 2005; Flynn et al. 2010) |

work to work. Nevertheless, Figure S3 illustrates the general trends implied by the constitutive relations for the three fiber systems. Collectively, these constitutive descriptions span a wide range of mechanical behaviors, allowing the model to represent collagen’s nonlinear stiffening response, elastin’s compliant yet compression-sensitive behavior, and actomyosin’s active contractile nature.

**S4. Multiscale Setup**

We adopt the multiscale network formulation approach of Mahutga et al. (Mahutga et al. 2023), which permits direct incorporation of DFN within a continuum mechanics framework. In this procedure, the stress contribution of the fiber network is evaluated through volume averaging theory, consistent with our earlier works (Chandran and Barocas 2007; Stylianopoulos and Barocas 2007). This theory gives us a feasible way of calculating the average Cauchy stress ${(\sigma}_{ij})$ in a representative volume $(V)$ as an integral of the local Cauchy stress $(\sigma_{ij}^{'})$ over the domain or a summation over the fiber elements $(e)$, expressed by the following equation:

$\begin{aligned} \sigma_{ij}=\frac{1}{V}\int\sigma_{ij}^{'}ⅆv \approx\frac{1}{V}\sum_{e} \sigma_{ij}^{e}v^{e}=\frac{1}{V}\sum_{e} \left( \frac{f^{ⅇ}n_{i}^{e}n_{j}^{e}}{a^{ⅇ}} \right)(a^{e}l^{e})= \frac{1}{V}\sum_{e} f^{e}l^{e}n_{i}^{e}n_{j}^{e} \#\left( 10 \right) \end{aligned}$

where $f^{e}$ is the constituent fiber force, $l^{e}$is the current fiber length, $n_{i}^{e}$ is the unit vector in the fiber’s axial direction, and $a^{e}$ is the deformed fiber cross-sectional area. This formulation enables the microstructural fiber response to be mapped onto the continuum stress field.

Under the assumption that inertial effects within the fiber network are insignificant, mechanical equilibrium is obtained by enforcing a force balance at every node, given the imposed deformation of the microstructural boundary. While several numerical strategies can be employed to solve this equilibrium problem, we adopt a damped Newton–Raphson iterative procedure based on the internal network Jacobian, defined as $J_{pq}=df_{p}^{n}/dx_{q}^{n}$. Here, $f_{p}^{n}$ denotes the force on node $n$ in direction $p$and $x_{q}^{n}$ denotes the corresponding nodal coordinate. We can derive the Jacobian analytically by summing the contributions from all fibers connected to each node,

$J_{pq}=\frac{df_{p}^{n}}{dx_{q}^{n}}=\sum_{e} \frac{d\left( f^{e}n_{p}^{en} \right)}{dx_{q}^{n}}=\sum_{e} \left( \frac{df^{e}}{dx_{q}^{n}}n_{p}^{en}+f^{e}\frac{dn_{p}^{en}}{dx_{q}^{n}} \right)$ $\left( 11a \right)$

which, using the chain rule, can be expressed as

Figure S3 Representative mechanical behavior of fiber constituents: (a) Collagen, (b) Elastin, and (c) Actomyosin. It should be noted that the quantitative values of these plots may vary across studies.

$J_{pq}=\sum_{e} \left( \frac{df^{e}}{d\lambda^{e}}\frac{d\lambda^{e}}{dl^{e}}\frac{dl^{e}}{dx_{q}^{n}}n_{p}^{n}+f^{e}\frac{dn_{p}^{en}}{dx_{q}^{n}} \right)=\sum_{e} \left( \frac{df^{e}}{d\lambda^{e}}\frac{dl^{e}}{dx_{q}^{n}}\frac{n_{p}^{en}}{L^{e}}+f^{e}\frac{dn_{p}^{en}}{dx_{q}^{n}} \right)$ $\left( 11b \right)$

where $n_{p}^{en}$ denotes the axial unit vector of fiber $e$ at node $n$, $f^{e}$ denotes the force in fiber $e$, and the fiber stretch is defined as $\lambda^{e}=l^{e}/L^{e}$, with$L^{e}$ and $l^{e}$denoting the reference and deformed fiber lengths, respectively. The deformed length $l^{e}$ is computed as the distance between the fiber’s two end nodes, whereas $n_{p}^{en}$ is calculated by dividing the vector difference between the two fiber end node positions by $l^{e}$. The quantity $\frac{df^{e}}{d\lambda^{e}}$, as already seen in **Section S3**, indicates fiber stiffness. The 1^st^ term in Eqn. (11b) captures the contribution of fiber elongation towards the Jacobian, whereas the 2^nd^ term accounts for alterations in fiber orientation associated with the rotation of an already stretched fiber. The resulting Jacobian is a 3N × 3N matrix, with $N$ indicating the number of network nodes. Because each fiber acts on only two nodes, not every node interacts with all other nodes. We pre-allocate the Jacobian matrix in the solution process and perform a loop over the fibers to accumulate the nodal values in the Jacobian by summing over the fibers.

We attain the solution for Newton update step via solving the linear system $J_{pq}\varepsilon_{q}=-R_{p}$. Here, $\varepsilon_{q}$ denotes the Newton loop step and $R_{p}$ denotes the nodal residual. Updated nodal coordinates are obtained using $x_{q}^{'}=x_{q}+S\varepsilon_{q}$, with $S\leq1$ serving as the damping parameter, keeping up the stability of Newton loop. $S$ = 0.25 is employed for the first five iterations, $S$ = 0.50 for the subsequent five iterations, and $S$ = 1 for all remaining iterations. We continue the loop until the residual’s norm goes below 1 fN, which is approximately four orders of magnitude smaller than the usual fiber forces. Notably, the Newton-Raphson algorithm utilized here becomes ill-posed when the fiber connectivity is less than 6. While the choice of boundary conditions influences the degree of non-affine deformation, it does not alter the volume-averaging relationship and can therefore be handled entirely at the microscale. Periodic boundary conditions are applied in this work, with the assumption that fiber crossing a boundary will have an identical fiber stretch on both sides of the boundary. This ensures the displacement continuity across the boundary. Again, as fiber stretch is preserved across the boundary, the corresponding forces are equal in magnitude and opposite in direction, thereby enforcing static equilibrium.

Both the stress response and the corresponding tangent elasticity tensor must be expressed as functions of deformation gradient for implementing the fiber networks (described in **Section S3**) in a finite element framework. We therefore derive the fourth-order material elasticity tensor $(\mathbb{C}_{IJKL})$, given by

$\mathbb{C}_{IJKL}=\frac{\partial S_{IJ}}{\partial E_{KL}}=2\frac{\partial S_{IJ}}{\partial C_{KL}}=2\frac{\partial S_{IJ}}{\partial F_{pQ}}\frac{\partial F_{pQ}}{\partial C_{KL}}$ $\left( 12a \right)$

which can be expanded as

$\mathbb{C}_{IJKL}=2\left( \frac{\partial S_{IJ}}{\partial F_{pQ}}\frac{\partial F_{pQ}}{\partial U_{MW}}\frac{\partial U_{MW}}{\partial C_{KL}} + \frac{\partial S_{IJ}}{\partial F_{pQ}}\frac{\partial F_{pQ}}{\partial R_{pM}}\frac{\partial R_{pM}}{\partial C_{KL}} \right)$ $\left( 12b \right)$

where$E_{KL}$ denotes the Green-Lagrange strain tensor, $S_{IJ}$ denotes the 2^nd^ Piola-Kirchoff (PK2) stress, $C_{KL}$ denotes the right Cauchy-Green tensor, and $F_{pQ}$denotes the deformation gradient. Using polar decomposition, the deformation gradient can be written as $F_{pQ}=R_{pW}U_{WQ}$, where $R_{pW}$ is the rotation tensor and $U_{WQ}$ is the right stretch tensor. With this decomposition, the right Cauchy-Green tensor is expressed as $C_{KL}=U_{WK}U_{WL}$.

Because the rotation tensor is independent of the right Cauchy-Green tensor, we obtain $\frac{\partial R_{pM}}{\partial C_{KL}}=\mathbf{0}$, and the 2^nd^ term in Eq. (12b) vanishes. The material elasticity tensor therefore reduces to

$\mathbb{C}_{IJKL}=2\frac{\partial S_{IJ}}{\partial C_{KL}}=2\frac{\partial S_{IJ}}{\partial F_{pQ}}\frac{\partial F_{pQ}}{\partial U_{MW}}\frac{\partial U_{MW}}{\partial C_{KL}}$ $\left( 13 \right)$

Again, the derivative of deformation gradient with respect to the right stretch tensor is obtained as $\frac{\partial F_{pQ}}{\partial U_{MN}}=R_{pW}\frac{\partial U_{WQ}}{\partial U_{MN}}=R_{pM}\delta_{QN}$, where $\delta_{QN}$ denotes the Kronecker delta. The derivative of the right stretch tensor with respect to the right Cauchy-Green tensor is given by the following purely kinematic 4^th^ order tensor:

$\frac{\partial U_{MN}}{\partial C_{KL}}=\left( \frac{\partial C_{KL}}{\partial U_{MN}} \right)^{-1}=\left( U_{MK}\delta_{LN} + U_{ML}\delta_{KN} \right)^{-1}$ $\left( 14 \right)$

Next, the Cauchy stress from Eq. (10) is first transformed into PK2 stress using the inverse deformation gradient $F_{Jk}^{-1}$ and the Jacobian, $J=\det(F)=dV/dV_{0}$. Assuming affine fiber deformation, such that $l^{e}n_{i}^{e}=L^{e}F_{iJ}N_{J}^{e}$, the approximate PK2 stress can be written as

$S_{IJ}=JF_{Jk}^{-1}\sigma_{kp}F_{Ip}^{-1}=\frac{V}{V_{0}}F_{Jk}^{-1}\left( \frac{1}{V}\sum_{e} f^{e}l^{e}n_{k}^{e}n_{p}^{e} \right)F_{Ip}^{-1}=F_{Jk}^{-1}\left( \frac{1}{V_{0}}\sum_{e} f^{e}L_{0}^{e}n_{k}^{e}N_{I}^{e} \right)$ $\left( 15 \right)$

where $L_{0}^{e}$ is the fiber rest length, while $N_{J}^{e}$ is the unit vector along the fiber axis in the reference configuration. Differentiating the PK2 stress with respect to the deformation gradient yields

$\frac{\partial S_{IJ}}{\partial F_{pQ}}=\frac{\partial F_{Jj}^{-1}}{\partial F_{pQ}}\left( \frac{1}{V_{0}}\sum_{e} f^{e}L_{0}^{e}n_{j}^{e}N_{I}^{e} \right)+F_{Jj}^{-1}\left( \frac{1}{V_{0}}\sum_{e} \frac{\partial\left( f^{e}n_{j}^{e} \right)}{\partial F_{pQ}}L_{0}^{e}N_{I}^{e} \right)$ $\left( 16 \right)$

where $\partial F_{Jj}^{-1}/\partial F_{pQ}=-F_{Jp}^{-1}F_{Qj}^{-1}$. Changes in fiber force due to deformation are approximated using a linearized fiber response for simplicity, where the tangent stiffness is defined as $k=df^{e}/d\varepsilon^{e}=df^{e}/d\lambda^{e}$. Under this assumption,

$$f^{e}n_{j}^{e}=k\left( \frac{l^{e}}{L_{0}^{e}} - 1 \right)n_{j}^{e}=k\left( \frac{l_{j}^{e}}{L_{0}^{e}} - n_{j}^{e} \right)=k\left( \frac{F_{jM}L_{0}^{e}N_{M}^{e}}{L_{0}^{e}} - n_{j}^{e} \right)=k\left( F_{jM}N_{M}^{e} - n_{j}^{e} \right)$$

$=k\left( F_{jM} - R_{jM} \right)N_{M}^{e}$	$\left( 17 \right)$

where the deformed fiber unit vector is expressed as a pure rotation of the reference fiber unit vector based on affine deformation assumption and polar decomposition. As $N_{M}^{e}$ is defined in the reference configuration, the derivative of $f_{j}^{e}$ with respect to the deformation gradient is given by

$$\frac{\partial f_{j}^{e}}{\partial F_{pQ}}=k\left( \frac{\partial F_{jM}}{\partial F_{pQ}} - \frac{\partial R_{jM}}{\partial F_{pQ}} \right)N_{M}^{e}=k\left( \delta_{jp}\delta_{MQ} - \frac{\partial R_{jM}}{\partial\left( R_{pW}U_{WQ} \right)} \right)N_{M}^{e}=k\left( \delta_{jp}\delta_{MQ} - U_{QW}^{-1}\delta_{jp}\delta_{WM} \right)N_{M}^{e}$$

$=k\left( N_{Q}^{e} - U_{QM}^{-1}N_{M}^{e} \right)\delta_{jp} (18)$

where $U_{MQ}^{-1}$ denotes the inverse right stretch tensor. Combining Eqns. (12)–(18) yields the material elasticity tensor, which is subsequently transformed into the spatial elasticity tensor for finite element implementation via

$c_{ijkl}=F_{iM}F_{jN}F_{kP}F_{lQ}\mathbb{C}_{MNPQ}$	$(19)$

However, the elasticity formulation derived previously (Eqns. (12)–(19)) assumes affine deformation of the fiber network and therefore does not incorporate non-affinity that may emerge during the solution of network equilibrium. To account for this non-affinity, we introduce a remapping procedure in which each network node is projected back to the reference configuration by employing the inverse deformation gradient

$X_{K}^{'B}=F_{Kv}^{-1}x_{v}^{B}$	$(20)$

where $X_{K}^{'B}$ denotes a new nodal coordinate for the equilibrated node $B$ mapped back to the reference configuration, while $x_{v}^{B}$ denotes the equilibrium coordinate of node $B$ in the current frame after the non-affine rearrangement of the network. The equilibrium configuration is obtained through Newton–Raphson iteration. In this context, $X_{K}^{'B}$ denotes the initial position of a node $B$ that, if subsequently deformed affinely, arrives in the same final position as the equilibrated node $x_{v}^{B}$ that started at $X_{K}^{B}$. This operation constructs an artificial network whose affine deformation reproduces the deformed state of the original network. This nodal remapping is essential for applying the approximate material elasticity derivations presented above in the finite element implementation, as it significantly refines the estimation of the elasticity tensor at the current deformation.

At the tissue scale, the material is represented as a constrained mixture of the DFN and an isotropic bulk matrix, modeled as a compressible neo-Hookean solid accounting for the non-fibrillar tissue components that stabilize the network and limit compressibility. The strain energy density function of the bulk matrix is given by the following expression:

$W=\frac{\mu}{2}(I_{1}-3)-\mu\ln(J)+\frac{\lambda}{2}(\ln J)^{2}$ $\left( 21 \right)$

where $\mu$is the second Lame parameter, $I_{1}$is the first invariant of the right Cauchy–Green tensor, $\lambda$ is the first Lame parameter, and $J$ is the determinant of the deformation gradient. The derivation of the stress and elasticity tensors for this constitutive equation is included in the FEBio theory manual (Maas et al. 2020). The homogenized tissue stress is then attained by superposing the matrix contribution with the volume-averaged stress of the DFN. In practice, this approach allows direct coupling between fiber-level mechanics and tissue-level finite element analyses.

**References:**

Aaron BB, Gosline JM (1981) Elastin as a random‐network elastomer: A mechanical and optical analysis of single elastin fibers. Biopolymers 20:1247–1260. https://doi.org/10.1002/bip.1981.360200611

Chandran PL, Barocas VH (2007) Deterministic Material-Based Averaging Theory Model of Collagen Gel Micromechanics. Journal of Biomechanical Engineering 129:137–147. https://doi.org/10.1115/1.2472369

Flynn BP, Bhole AP, Saeidi N, et al (2010) Mechanical Strain Stabilizes Reconstituted Collagen Fibrils against Enzymatic Degradation by Mammalian Collagenase Matrix Metalloproteinase 8 (MMP-8). PLoS ONE 5:e12337. https://doi.org/10.1371/journal.pone.0012337

Freed AD, Doehring TC (2005) Elastic Model for Crimped Collagen Fibrils. Journal of Biomechanical Engineering 127:587–593. https://doi.org/10.1115/1.1934145

Gacek E, Mahutga RR, Barocas VH (2023) Hybrid discrete-continuum multiscale model of tissue growth and remodeling. Acta Biomaterialia 163:7–24. https://doi.org/10.1016/j.actbio.2022.09.040

Gosline J, Lillie M, Carrington E, et al (2002) Elastic proteins: biological roles and mechanical properties. Philosophical Transactions of the Royal Society of London Series B: Biological Sciences 357:121–132. https://doi.org/10.1098/rstb.2001.1022

Maas S, Rawlins D, Weiss J, Ateshian G (2020) FEBio theory manual. Salt Lake City

Mahutga RR, Badal RM, Barocas VH, Alford PW (2025) A multiscale discrete fiber model of failure in heterogeneous tissues: Applications to remodeled cerebral aneurysms. Journal of Biomechanics 178:112343. https://doi.org/10.1016/j.jbiomech.2024.112343

Mahutga RR, Barocas VH, Alford PW (2023) The non-affine fiber network solver: A multiscale fiber network material model for finite-element analysis. Journal of the Mechanical Behavior of Biomedical Materials 144:105967. https://doi.org/10.1016/j.jmbbm.2023.105967

Rachev A, Hayashi K (1999) Theoretical Study of the Effects of Vascular Smooth Muscle Contraction on Strain and Stress Distributions in Arteries. Annals of Biomedical Engineering 27:459–468. https://doi.org/10.1114/1.191

Stylianopoulos T, Barocas VH (2007) Volume-averaging theory for the study of the mechanics of collagen networks. Computer Methods in Applied Mechanics and Engineering 196:2981–2990. https://doi.org/10.1016/j.cma.2006.06.019
